# Modulation of Tomato Response to *Rhizoctonia solani* by *Trichoderma harzianum* and Its Secondary Metabolite Harzianic Acid

**DOI:** 10.3389/fmicb.2018.01966

**Published:** 2018-08-30

**Authors:** Gelsomina Manganiello, Adriana Sacco, Maria R. Ercolano, Francesco Vinale, Stefania Lanzuise, Alberto Pascale, Mauro Napolitano, Nadia Lombardi, Matteo Lorito, Sheridan L. Woo

**Affiliations:** ^1^Department of Agricultural Sciences, University of Naples Federico II, Portici, Italy; ^2^National Research Council, Institute for Sustainable Plant Protection, Portici, Italy; ^3^Task Force on Microbiome Studies, University of Naples Federico II, Naples, Italy; ^4^Department of Pharmacy, University of Naples Federico II, Naples, Italy

**Keywords:** *Trichoderma harzianum*, secondary metabolites, tomato, biological control, beneficial microbes, resistance response, DEGs, harzianic acid

## Abstract

The present study investigated the transcriptomic and metabolomic changes elicited in tomato plants (*Solanum lycopersicum* cv. Micro-Tom) following treatments with the biocontrol agent *Trichoderma harzianum* strain M10 or its purified secondary metabolite harzianic acid (HA), in the presence or the absence of the soil-borne pathogen *Rhizoctonia solani*. Transcriptomic analysis allowed the identification of differentially expressed genes (DEGs) that play a pivotal role in resistance to biotic stress. Overall, the results support the ability of *T. harzianum* M10 to activate defense responses in infected tomato plants. An induction of hormone-mediated signaling was observed, as shown by the up-regulation of genes involved in the ethylene and jasmonate (ET/JA) and salicylic acid (SA)-mediated signaling pathways. Further, the protective action of *T. harzianum* on the host was revealed by the over-expression of genes able to detoxify cells from reactive oxygen species (ROS). On the other hand, HA treatment also stimulated tomato response to the pathogen by inducing the expression of several genes involved in defense response (including protease inhibitors, resistance proteins like CC-NBS-LRR) and hormone interplay. The accumulation of steroidal glycoalkaloids in the plant after treatments with either *T. harzianum* or HA, as determined by metabolomic analysis, confirmed the complexity of the plant response to beneficial microbes, demonstrating that these microorganisms are also capable of activating the chemical defenses.

## Introduction

The complex network of interactions established by plants with the rhizosphere microbiota greatly affect crop health and fitness (Raaijmakers et al., 2009; Berendsen et al., 2012). The demonstrated benefits include stimulation of plant growth, enhanced pathogen control, improved abiotic stress resistance, increased nutrient availability and uptake, higher yield and better product quality (Berg, 2009; Berg et al., 2014; Trivedi et al., 2016; Martínez-Medina et al., 2017; Pascale et al., 2017). Plant-microbe interactions also include cases that may be detrimental, compromising plant growth and health, although the establishment of a disease status is often counteracted by the presence of a beneficial soil microbial community that can hinder infection by disease pathogens (Matos et al., 2005; Walters et al., 2008). Mycoparasitic fungi such as *Trichoderma* and *Gliocladium* have antagonistic activity on many soilborne pathogens (including *Verticillium, Sclerotinia, Rhizoctonia, Fusarium, Pythium*) due to a variety of mechanisms, including: parasitism, competition for nutrients and space, antibiosis, and production of lytic enzymes, as well as the ability to trigger the induction of systemic resistance (ISR) (Harman et al., 2004b; Lorito et al., 2010; Doornbos et al., 2012; Hermosa et al., 2014; Amira et al., 2017; Nawrocka et al., 2018).

To date, disease management in agriculture has relied mainly on the application of chemical pesticides. Even though this protection strategy may be efficient and in many cases cost-effective, it poses serious risks to human health and the environment (Leach and Mumford, 2008). With the implementation of Directive 2009/128/EC, the European Community promotes specific actions to support the establishment of sustainable agriculture through the reduced use of chemical pesticides in favor of alternative non-chemical products, and the promotion of integrated pest management (IPM).

Therefore, the development of bio-based strategies to enhance crop production and food safety has become a cutting-edge research topic in biological and agricultural sciences. Numerous beneficial microorganisms are already in use as active ingredients in bio-pesticides, bio-fertilizers, bio-stimulants, plant growth enhancers and soil integrator formulations (Verma et al., 2007; Harman et al., 2010; Lorito et al., 2010; Woo et al., 2014). Many of them are based on endophytic or plant-root associated fungi belonging to the genus *Trichoderma* due to the ability of these microbes to control different phytopathogens and exert a number of positive effects on crops. Some *Trichoderma* spp. are characterized by competency for the rhizosphere environment, being able to extensively colonize the root system starting from the germinating seed (Yedidia et al., 1999; Harman, 2000; Harman et al., 2004a). Production of auxins or auxin–like compounds by the fungus stimulates root formation and development, thus expanding the area available for colonization (Vinale et al., 2008, 2012; Contreras-Cornejo et al., 2009; Hermosa et al., 2012).

The plant-*Trichoderma* interaction may be classified as a facultative symbiosis characterized by the establishment of reciprocal advantages. The fungus occupies a strategic ecological niche, obtains nutrients from the host, and in turn provides beneficial effects to the plant that include direct protection to pathogen attack, solubilization of nutrients, improvement of growth and vigor, plus the “alerting” (priming) of defense response (Harman et al., 2004b; Vargas et al., 2009, 2011; Shoresh et al., 2010). Furthermore, tomato plants subjected to biotic and/or abiotic stresses release specific root exudates that act as chemo-attractants for *Trichoderma*, which are able to recognize “help” signals and grow toward the stressed plants (Lombardi et al., 2018). Root colonization by *Trichoderma* induces root-hair development and defense responses, generates substantial changes in diverse metabolic pathways and triggers the expression of genes involved in plant defense mechanisms mainly associated to jasmonic acid- (JA) and ethylene- (ET) dependent signaling pathways (Vinale et al., 2008; Contreras-Cornejo et al., 2015; Martínez-Medina et al., 2017). In the case of *Arabidopsis*, colonization by *Trichoderma*, prior to infection by biotrophic or necrotrophic phytopathogens, activated a priming status that was able to systemically enhance resistance (Salas-Marina et al., 2011; Hermosa et al., 2013). Moreover, some *Trichoderma* strains can produce microbe-associated molecular patterns (MAMPs) that induce plant defense responses including the production of elicitors such as a xylanase (Xyn2/Eix) in tomato and tobacco, an endopolygalacturonase in *Arabidopsis thaliana*, and a swollenin (TasSwo) in cucumber (Rotblat et al., 2002; Brotman et al., 2008; Morán-Diez et al., 2009).

*Trichoderma* spp. are important producers of secondary metabolites (SM) that provide selective advantages in processes like competition, symbiosis, metal transport, growth differentiation, signaling, mycoparasitic activity etc. (Sivasithamparam and Ghisalberti, 1998; Woo et al., 1999; Harman et al., 2004b; Reino et al., 2008; Lorito et al., 2010). Harzianic acid (HA), a tetramic acid produced by *Trichoderma harzianum* M10 strain, demonstrated remarkable biological properties, including plant growth promotion, antimicrobial activity against different pathogens such as *Pythium irregulare, Sclerotinia sclerotiorum*, and *Rhizoctonia solani*, plus an ability to chelate soil iron (Fe3^+^) thus facilitating its uptake by the plant (Vinale et al., 2013).

In many instances, the application of a purified compound, such as a SM, was found to produce effects comparable to those obtained by treating the crop with the living microbe (Vinale et al., 2008; Pascale et al., 2017). The use of these naturally-derived compounds in alternative to, or in combination with the living microbe may contribute to developing novel plant protection products and biofertilizers that may be more effective and reliable when applied to a variety of crops and environment conditions.

The effects of living *Trichoderma* on the plant transcriptome, metabolome, and proteome have been extensively studied (Marra et al., 2006; Chacón et al., 2007; Lorito et al., 2010; Morán-Diez et al., 2012, 2015; Mendoza-Mendoza et al., 2018). However, the role of purified secondary metabolites produced by the same fungus in the interaction with the plant has not yet been fully clarified.

In order to dissect the molecular basis of defense responses and induction of resistance mechanisms activated during *Trichoderma*-plant interactions, we tested and compared the effect of *T. harzianum* strain M10 and its secondary metabolite HA. The patterns of differentially expressed genes (DEGs) and differentially abundant plant secondary metabolites were determined by analyzing transcriptomic and metabolomic changes occurring in tomato plants colonized by *T. harzianum* M10 or treated with HA, in the presence or the absence of the soilborne pathogen *R. solani*.

## Materials and methods

### Fungal strains and plant material

*Trichoderma harzianum* strain M10, isolated from composted hardwood bark in Western Australia, was maintained and routinely propagated to complete sporulation on Potato Dextrose Agar (PDA; SIGMA, St Louis, MO) slants at room temperature and sub-cultured monthly. Ten 7-mm diameter plugs of actively growing M10 were obtained from the margins of PDA cultures and inoculated to 5-L conical flasks containing 2 L of sterile potato dextrose broth (PDB; SIGMA). Cultures were incubated for 30 days at 25°C without agitation, then the fungal biomass was removed and the liquid culture was filtered through Whatman paper (No. 4, Brentford, UK) under vacuum. For the production of HA, the fungal culture filtrate was extracted exhaustively with ethyl acetate (EtOAc). The purification and biochemical characterization of HA was carried out as previously reported (Vinale et al., 2009, 2013, 2014).

Fresh conidia were collected in sterile water from sporulating fungal cultures of M10 grown for 7 days on PDA at 25°C. Tomato (*Solanum lycopersicum* cv. Micro-Tom) seeds were surface sterilized using 70% (v/v) ethanol for 2 min, followed by 2% (v/v) sodium hypochlorite solution for 2 min, rinsed well with sterile distilled water, and left to air-dry.

The soilborne fungal pathogen *R. solani* was isolated from naturally infected tomato seedlings. Cultures were maintained on PDA for 1 week at 25°C, then ten 7-mm diameter mycelia plugs were inoculated to 5-L conical flasks containing 2 L of sterile PBD. The culture was incubated for 15 days at 25°C, with orbital agitation at 150 rpm, then paper filtered under vacuum to harvest the biomass.

### Plant treatments

The experiment was divided into two blocks based on the absence (Block 1) or the presence (Block 2) of the pathogen. Thirty tomato seeds were employed for each treatment and sown in 500-mL pots containing sterile soil, with three independent biological replicates used for each treatment.

Treatments without the *Rhizoctonia* pathogen were comprised of:

- Plant-H_2_O [P]. Seeds were treated with water and plants only received water for the duration of the experiment.
- Plant-M10 [P+T]. Seeds were coated with *T. harzianum* M10 spores (1 × 10^7^ spores/mL) in water, stirring to uniformly coat the entire surface, left to air-dry, and stored at 4°C until planting in sterile soil.
- Plant-HA [P+HA]. Seeds were treated with water, and sown in sterile soil, then 30 days after emergence, all plants were treated with a 50 mL volume of the HA solution [10^−6^ M] by foliar spray.

Treatments with the *Rhizoctonia* pathogen were comprised of:

- Plant-*R. solani* [P+R]. Seeds were treated with water and sown in sterile soil. One month after the emergence the soil was inoculated with 50 mL of *R. solani* biomass homogenized in water (2% w/v).
- Plant-M10-*R. solani* [P+T+R]. Seeds were coated with *T. harzianum* M10 spores (1 × 10^7^sp/mL) and sown in sterile soil; then, 1 month after emergence, the soil was infected with *R. solani* biomass (2% w/v).
- Plant-HA-*R. solani* [P+HA+R]. Seeds were treated with water, and sown in sterile soil, then 30 days after emergence, were treated with a 50 mL volume of the HA solution [10^−6^ M] by foliar spray. Then 24 h following the treatment, the soil was infected with the *R. solani* biomass (2% w/v).

All plants were placed in a growth chamber under controlled conditions, temperature (25°C), RH 70% and photoperiod (16 h of light/8 dark). Whole plants were harvested at the same time, equivalent to 48 h after pathogen inoculum (if present), frozen in liquid nitrogen, ground to a fine powder and stored at −80°C until further processing.

### Biocontrol assay of *Rhizoctonia solani*

Biocontrol assays were conducted as described in the above section for Block 2, with 1-month old plants infected with *R. solani*, to test the effect of the *Trichoderma* M10 (seed coating) and the HA (foliar spray) on disease development. Each treatment consisted of 15 plants, in 3 replicates (total of 45 plants,) with the experiment arranged in random block design. The disease development was evaluated every 24 h for a 6 day period after pathogen inoculation, and the disease incidence was determined as the percentage of plants demonstrating symptoms of *R. solani* crown rot infection.

### RNA extraction and microarray analysis

Each treatment consisted of 20 plants. Total RNA was extracted and purified using PureLink® RNA Mini Kit (Ambion Inc., Austin, TX) from a pool of equal amounts of the powdered vegetative material obtained from the 20 tomato plants of each treatment. Removal of genomic DNA was performed by digestion with DNase I, Amplification Grade (Invitrogen, USA). Total RNA quantity and quality was assessed using NanoDrop 1000 (Thermo Scientific, Waltham, MA). The quality of RNA is a critical factor in hybridization performance, therefore only RNA samples with 230/260 and 260/280 ratios >2 were used.

Total RNA (2 μg) was amplified and labeled using the RNA ampULSe kit (Kreatech Biotechnology, Amsterdam, Netherlands). Microarray hybridization was performed with 4 μg of Cy5 labeled aRNA inoculated at 45°C for 16 h in the Pre-hybridization Solution. After incubation, arrays were washed with different Washing Solutions at 45°C (6X SSPET Wash, 5 min with gentle rotation; 3X SSPET Wash, 1 min; 0.5X SSPET Wash, 1 min; PBST Wash, 1 min) and two times with PBS Wash at room temperature for 1 min. Labeled aRNA was hybridized to the TomatoArray 2.0 (CombiMatrix microarray platform, Italy), then analyzed using a Perkin Elmer ScanArray 4000 XL scanner. Images were acquired with ScanArray Express Microarray Analysis System (Version 4.0) software. The samples analyzed are reported in Table 1A. Microarrays were stripped for reuse (each chip was used up to three times) using the CombiMatrix CustomArray Stripping Kit according to the manufacturer's instructions. Two biological replicates were employed to evaluate the differential gene expression.

**Table 1:** Plant material used in the transcriptomic and comparative analysis obtained by treating tomato (P) with Trichoderma M10 (T) or harzianic acid (HA), and the pathogen Rhizoctonia (R) pathogen.

**No Pathogen**	**With Pathogen**
**(A) HYBRIDIZATION**	
Plant-H_2_O [P]	Plant + *R. solani* [P+R]
Plant + *T. harzianum* M10 [P+T]	Plant + *T. harzianum* M10 + *R. solani* [P+T+R]
Plant + Harzianic Acid [P+HA]	Plant + HA + *R. solani* [P+HA+R]
**(B) COMPARISONS**	
	Plant + M10 + *R. solani* [P+T+R] vs. Pathogen [P+R]
Plant-M10 [P+T] vs. Plant-H_2_O [P]	Plant + HA + *R. solani* [P+HA+R] vs. Pathogen [P+R]
	Plant + HA + *R. solani* [P+HA+R] vs. Plant + M10 + *R. solani* [P+T+R]

*A, Sample conditions used in tomato CombiMatrix microarray hybridizations; B, Combinations of treatments used in the comparison analyses*.

### Chip annotation and bioinformatic analysis

Transcriptome analysis was performed using the 90 K TomatoArray 2.0 chip with probes synthesized at the Functional Genomics Centre of the University of Verona using the CombiMatrix platform. The TomatoArray 2.0 chip has 25,789 oligonucleotide probes of 35-40-mer, randomly distributed in triplicate across the array. Each probe was constructed to be complementary to a Tentative Consensus sequence (TC), resulting from DFCI Tomato Gene Index Release 12.0. Eight bacterial oligonucleotides provided by CombiMatrix, as well as 40 probes of *Bacillus anthracis, Haemophilus ducreyi*, and *Alteromonas*, were included in the chip design as negative controls.

The TCs reported in Tomato Gene Index Release 12.0 (ftp://occams.dfci.harvard.edu/pub/bio/tgi/data/Solanum_lycopersicum/) were mapped to tomato chromosomes, blasting the probes used to design the chip against the Database WGS Chromosomes (SL 2:50) of Sol-Genomics Network (https://solgenomics.net/). Only the data with an e-value between 0 and 1.0^−6^ were considered for further analysis. Each Solyc ID obtained from TCs was removed from the tomato annotation files ITAG 2.40, thus allowing the annotation of the complete chip. The DEGs were identified using the software R (R Core Team, 2013 http://www.R-project.org/). In particular, the acquired CombiMatrix arrays were analyzed through Bioconductor package (Gentleman et al., 2004). The software MapMan ORC 3.1 (Thimm et al., 2004) was used to map the DEGs for all the experimental conditions, and KEGG database (http://www.genome.jp/kegg/) was used for further reconstruction of the biosynthetic pathways.

### Expression profiling by qPCR

One μg of purified total RNA was used as a template for first-strand cDNA synthesis using SuperScript® III Reverse Transcriptase (Invitrogen). Seven genes for each treatment were amplified to validate the microarray results. These genes were selected from DEGs lists obtained for each condition. *S. lycopersicum* primer sequences were designed using the Primer3 online tool (http://primer3.ut.ee/) and are listed in (Table S1). All samples were normalized to *apha*-*tubulin* as reference housekeeping gene. Gene transcript levels were measured using Power SYBR® Green PCR Master Mix (Applied Biosystems®) on an ABI PRISM 7900HT sequence detection system (Applied Biosystems®). Data were analyzed with 7900 V 2.0.3 evaluation software (Applied Biosystems®). The relative quantitative expression was determined using the 2^−ΔΔCT^ method (Livak and Schmittgen, 2001).

### Plant metabolome analysis

Ten mg of ground tomato from the infected plants treated with *Trichoderma* and HA were used for metabolite extraction in 0.8 mL of 20% methanol in water. Samples were centrifuged (10 min at 16,100 g, 4°C), and the supernatant was filtered through a 0.2 μm polyvinylidine fluoride (PVDF) filter (Chromacol, Welwyn Garden City, UK). Sample extracts (7 μl) were loaded onto a Poroshell 120EC-C18 1.8 Pm, 2.1 × 5 mm reverse phase analytical column (Agilent Technologies, Palo Alto, USA) for metabolite profiling performed in a 6540 UHD Accurate Mass QTOF LC-MS/MS mass spectrometer (Agilent Technologies, Palo Alto, USA), in MS mode coupled to a 1200 series Rapid Resolution + HPLC. Mobile phases consisted in water (Cromasolv® Plus, LC-MS-Sigma) with 0.1% LC-MS grade formic acid (A) and acetonitrile (Cromasolv® Plus, LC-MS-Sigma) with 0.1% LC-MS grade formic acid (B). The separation was carried out by the following gradient: 0 min−5% B; 12 min−100% B; 15 min−100% B; 17 min−95% B; 20 min−95% B, 2 min post-time. The flow rate was 0.6 ml min^−1^ and the column temperature was held at 35°C. The source conditions for electrospray ionization were the following: nitrogen gas temperature was 350°C with a drying gas flow rate of 11 L min^−1^ and a nebulizer pressure of 45 psig. The fragmentor voltage was 180 V and skimmer voltage 45 V. The range acquisition of TOF spectra was from 50 to 1,600 *m/z* with an acquisition rate value of three spectra sec^−1^. The data were collected in positive ion mode. The mass spectra were submitted to the Agilent MassHunter Profinder software and then to MassProfile Professional Software to compute the annotation and statistical analysis.

### Statistical analysis

Data from biocontrol experiments were subjected to statistical analysis using “Agricolae” package of R software, from the R project (www.r-project.org) of a repository CRAN, de Mendiburu and de Mendiburu (2017). Statistical analysis was performed by ANOVA followed by Least Significant Difference (LSD) *post-hoc* test using Bonferroni correction (*p* < 0.05).

The DEGs were identified using the software R (R Core Team, 2013 http://www.R-project.org/). In particular, the acquired CombiMatrix arrays were analyzed through Bioconductor package (Gentleman et al., 2004). The software MapMan ORC 3.1 (Thimm et al., 2004) was used to map the DEGs for all the experimental conditions. The fluorescence data were normalized applying the quantile normalization and the expression value was estimated using the empirical Bayesian approach (Wu et al., 2004). Subsequently, data were filtered for an adjusted *p* ≤ 0.05 and a Fold Change (FC) ≥ ±1, in order to obtain the complete list of DEGs. The comparison analysis used to identify DEGs in each type of interaction is reported in Table 1B. The software MapMan ORC 3.1 (Thimm et al., 2004) was used to map the DEGs for all the experimental conditions.

Enrichment analysis of the complete set of DEGs obtained was carried out using the binomial statistics tool to determine over- or under- representation of PANTHER or GO ontology classification categories (http://www.geneontology.org/). Each DEGs list was compared to a reference list using the binomial test (Cho and Campbell, TIGs 2000) for each Gene Ontology category. The settings for this analysis were as below:

- Annotation data set: PANTHER GO-slim.
- Reference gene list: All genes in PANTHER database for the selected organism (*S. lycopersicum*).
- Correction: Bonferroni.

The data from mass-spectrometry were analyzed considering a minimum absolute abundance of 5,000 counts, minimum number of ions 2. FC was calculated using the infected plant [P+R] as a control and only entities with −2 < FC > 2 were selected. Subsequently, the entities filtered were identified using ID browser identification associated to a Metlin Library (Agilent). A total of 25 compounds were found. The entities list was loaded in the clustering analysis tool using a hierarchical algorithm. The map was built on the three analyzed conditions [P+R, P+HA+R, P+T+R] using normalized log FC values, the Euclidean metric distance and the Ward's Linkage Rule.

## Results

### Biocontrol assay of *R. solani*

**Figure 1:**
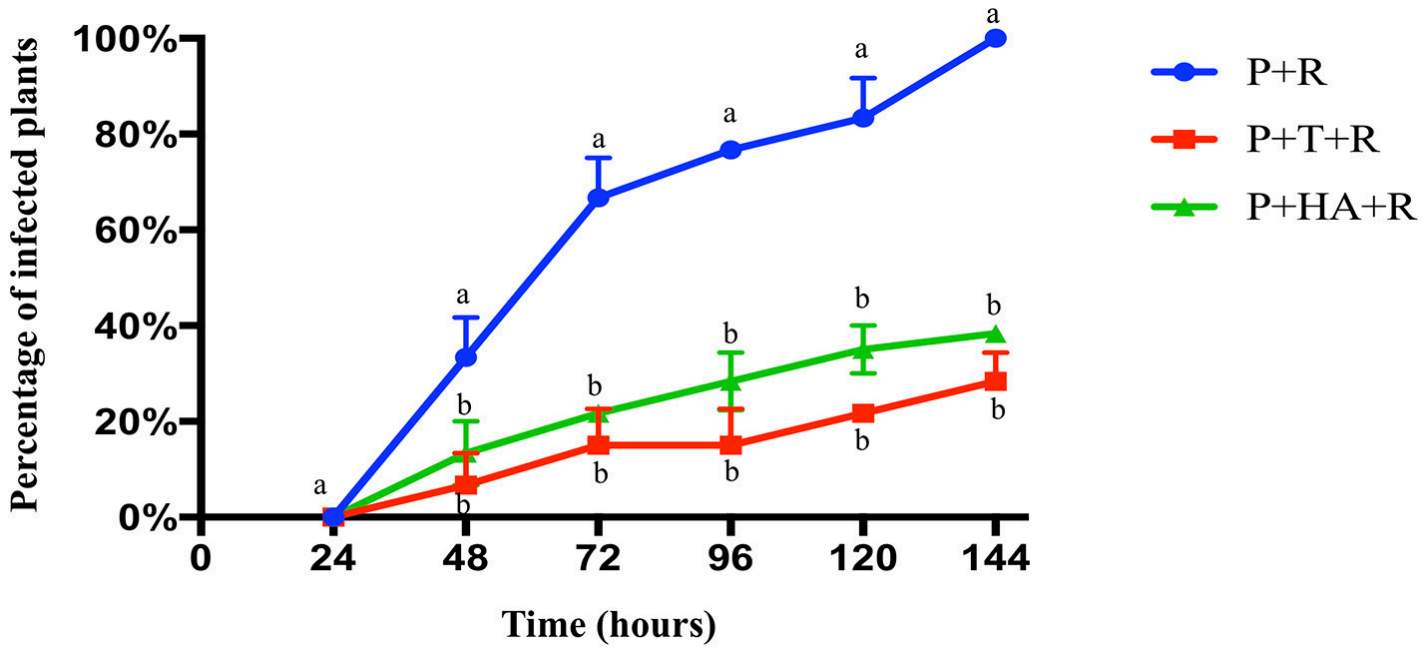
Biocontrol activity of *T. harzianum* M10 and harzianic acid on the development of *R. solani* disease symptoms on tomato. Pathogen attack was noted by the appearance of lesions on plant stem. Plant treatments included: P+R = *R. solani*- infected plants (blue); P+T+R = *Trichoderma* + *R. solani* (red); and P+HA+R = harzianic acid + *R. solani* (green). Data are presented as the mean of three independent experiments ± SEM. Different letters indicate significantly different values after two-way ANOVA followed by Bonferroni correction (*p* < 0.05) for each time point measurement.

The biocontrol activity of *T. harzianum* M10 and HA against *R. solani* on tomato was evaluated as the percentage of infected plants observed at 7 different time points (Figure 1). The first *R. solani* disease symptoms, indicated with the appearance of brown to reddish lesions on the stem/crown zone, appeared 24 h post-inoculum (HPI) in more than 30% of the *R. solani* infected plants, and a significant increase (77%) was noted after 48 h. Plants treated with M10 (P+T+R) or HA (P+HA+R) showed a much slower progression of disease development, with a reduction in infection at all measured time points significantly different in comparison to control (P+R). At the end of the experiment, only 35% of M10 and 38% of HA-treated plants were infected, in comparison to 100% infection rate observed in the control group. Interestingly, M10 and HA treatments demonstrated comparable efficacy in controlling disease (Figure 1). This experiment was useful to determine the optimal time point for the collection of samples to use in the following transcriptomic analysis. The 48 HPI time point was selected because it allowed the collection of a sufficient number of infected but still healthy plants for each experimental group, which is critical for obtaining a good quality RNA suitable for transcriptomic analysis.

### Plant-*Trichoderma harzianum* M10 interaction

**Figure 2:**
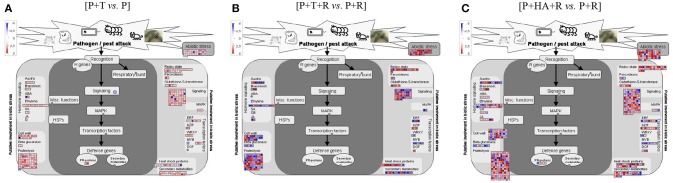
Diagrammatic representation of DEGs involved in “Biotic stress” category as visualized by MapMan. **(A)** [P+T vs. P]: plant treated with *Trichoderma* compared to Control plants (water); **(B)** [P+T+R vs. P+R]: infected plants treated with *Trichoderma* compared to infected plants (*R. solani*); **(C)** [P+HA+R vs. P+R]: infected plants treated with HA compared to infected plants (*R. solani*). Each gene is symbolized by a box, the gene expression level is color-encoded (red = up-, blue = down-regulation). Genes with experimental indication of involvement in the biotic stress are grouped in the main central panel (colored in dark gray), while genes and pathways that are putatively involved in biotic stress pathway are shown on the left and right sides (colored in light gray).

**Figure 3:**
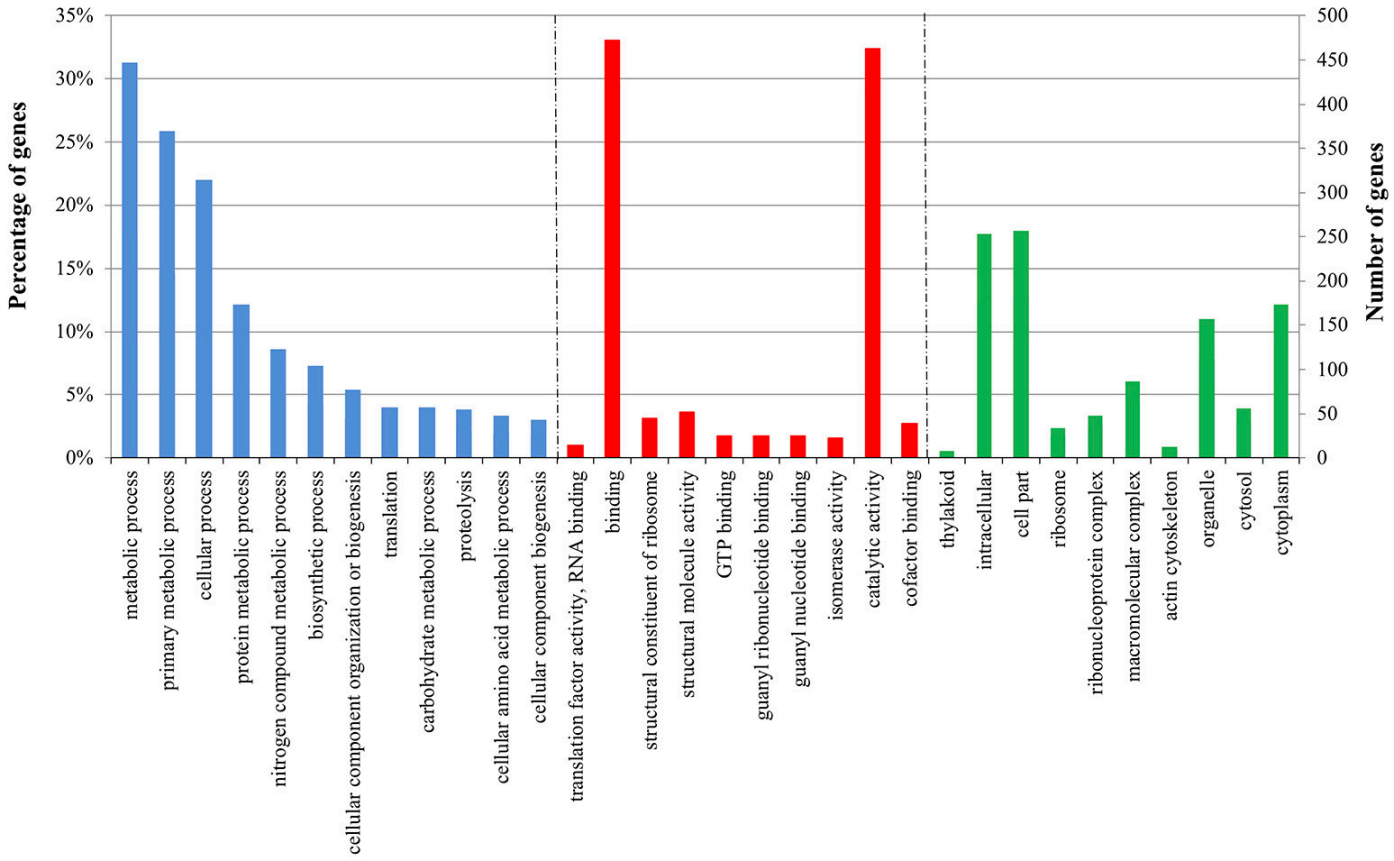
Enriched Gene Ontology (GO) terms distribution of tomato DEGs in plant-*Trichoderma* [P+T vs. P] interaction 30 days post-inoculum. The GO terms displayed along the X-axis are grouped in three GO categories: Biological Process (blue); Molecular function (red); and Cellular component (green). The values on the Y-axis indicate tomato genes in each GO term represented as: on the left, the percentage of total genes; and on the right, the number of genes detected in each GO term.

To monitor the global gene expression changes in tomato after colonization with *Trichoderma* M10 [P+T vs. P], a microarray analysis was performed 30 days post-inoculum (DPI). The complete DEG lists of each analyzed condition are reported in Supplementary Material Data Sheets 1–3. A total of 1,227 DEGs were observed, of which 1,142 were up-regulated and 85 down-regulated (Table 2). To identify the processes in which these genes are involved, DEGs obtained from each comparison were mapped to the main metabolic pathways using the MapMan software. The DEGs involved in the “Cellular Function” category, which represents an overview of all the pathways identified, were obtained for each of the following interaction comparisons [P+T vs. P], [P+T+R vs. P+R], and [P+HA+R vs. P+R] (Table 3). Among the categories noted in the [P+T vs. P] comparison, the most prevalent genes were involved in “protein synthesis/degradation” (231 genes), “RNA metabolism” (129 genes), “redox reactions” (24 genes), “signaling” (79 genes), “photosynthesis” (32 genes), “cell wall synthesis/degradation” (17 genes), “secondary metabolism” (28 genes), and “stress (biotic/abiotic)” (50 genes) (Table 3A). Based on MapMan prediction, several genes mapped to these categories were found to be putatively involved in biotic stress-related processes (Figure 2A). Over-expression of genes associated to ethylene production was predominant among hormone signaling pathways, and a general over-expression of genes coding for transcription factors such as ERF, bZIP, WRKY, and MYB was also observed. Several genes directly involved in pathogen recognition and plant defense were up-regulated; among them 2 pathogenesis related (PR) proteins coding transcripts and 1 gene involved in signaling (Enhanced disease susceptibility 1-*EDS1*). Furthermore, numerous genes involved in isoprenoids, phenylpropanoids, flavonoids, alkaloids and aromatic amino acids biosynthesis were over-expressed. Figure 3 presents enriched Gene Ontology (GO) terms obtained by Singular Enrichment Analysis (SEA) of the 1227 DEGs. The “Biological process” GO category contained 12 GO terms, while “Molecular function” and “Cellular component” categories contained 10 GO terms each. The majority of the tomato DEGs were associated to “cellular process,” “metabolic process,” “primary metabolic process,” “binding,” “catalytic activity”; these terms were found dominant (Figure 3). The transcriptomic analysis of HA-treated plants compared to untreated plants ([P+HA]) did not reveal any DEGs.

**Table 2:** Number of differentially expressed genes (DEGs) up-regulated (Up) and down-regulated (Down) in tomato plants treated with *Trichoderma, HA* and/or *Rhizoctonia*.

**Comparisons**	**DEGs**	**Up**	**Down**
[P+T vs. P]	1,227	1,142	85
[P+HA vs. P]	–	–	–
[P+T+R vs. P+R]	1,142	630	512
[P+HA+R vs. P+R]	2,317	1,456	861

*[T+P vs. P], plants treated with Trichoderma compared to control plants (water); [P+T+R vs. P+T], pathogen-infected plants treated with Trichoderma compared to untreated infected plants; [P+HA+R vs. P+R], infected plants treated with HA compared to untreated infected plants*.

**Table 3:** List of pathways obtained from Cellular Function overview (MapMan category) with the related number of DEGs involved.

**A**		**B**		**C**	
**Pathway name**	**No. of genes**	**Pathway name**	**No. of genes**	**Pathway name**	**No. of genes**
Photosynthesis	32	Photosynthesis	29	Photosynthesis	44
Major carbohydrate metabolism	12	Major carbohydrate metabolism	5	Major carbohydrate metabolism	12
Minor carbohydrate metabolism	8	Minor carbohydrate metabolism	4	Minor carbohydrate metabolism	10
Glycolisys	10	Glycolisys	5	Glycolisys	11
Fermentation	2	Fermentation	4	Fermentation	7
Gluconeonese/glyoxylate cycle	1	Gluconeonese/glyoxylate cycle	2	Gluconeonese/glyoxylate cycle	3
OPP.oxidative PP.6-phosphogluconolactonase	4	OPP.oxidative PP.6-phosphogluconolactonase	2	OPP.oxidative PP.6-phosphogluconolactonase	9
Tricarboxylic acid (TCA) cycle	6	Tricarboxylic acid (TCA) cycle	9	Tricarboxylic acid (TCA) cycle	23
Mitochondrial electron transport/ATP synthesis	9	Mitochondrial electron transport/ATP synthesis	12	Mitochondrial electron transport/ATP synthesis	23
		Synthesis	12	Synthesis	18
Cell wall synthesis/degradation	17	Cell wall synthesis/degradation	27	Cell wall synthesis/degradation	54
Lipid metabolism	23	Lipid metabolism	24	Lipid metabolism	49
N-metabolism	5	N-metabolism	3	N-metabolism	4
Amino acid metabolism	27	Amino acid metabolism	21	Amino acid metabolism	44
				S-assimilation	2
Metal handling	2	Metal handling	4	Metal handling	9
Secondary metabolism	28	Secondary metabolism	15	Secondary metabolism	33
Hormone metabolism	28	Hormone metabolism	30	Hormone metabolism	63
Co-factor and vitamin e metabolism	3	Co-factor and vitamin e metabolism	5	Co-factor and vitamin e metabolism	6
Tetrapyrrole synthesis	9	Tetrapyrrole synthesis	7	Tetrapyrrole synthesis	12
Stress biotic/abiotic	50	Stress biotic/abiotic	42	Stress	93
Redox reactions	24	Redox reactions	16	Redox reactions	33
Polyamine metabolism	2	Polyamine metabolism	1	Polyamine metabolism	5
Nucleotide metabolism	5	Nucleotide metabolism	11	Nucleotide metabolism	21
Biodegradation of Xenobiotics	4	Biodegradation of Xenobiotics	3	Biodegradation of Xenobiotics	4
		C1-metabolism	5	C1-metabolism	6
Miscellaneous	67	Miscellaneous	55	Miscellaneous	121
RNA metabolism	129	RNA metabolism	142	RNA metabolism	284
DNA synthesis	21	DNA synthesis	25	DNA synthesis	43
Protein synthesis/degradation	231	Protein synthesis/degradation	241	Protein synthesis/degradation	505
Signaling	79	Signaling	49	Signaling	122
Cell cycle	41	Cell cycle	54	Cell cycle	83
		MicroRNA, natural antisense	1	MicroRNA, natural antisense	1
Development	29	Development	35	Development	59
Transport	52	Transport	50	Transport	109
Not assigned	287	Not assigned	223	Not assigned	454

*A, [P+T vs. P]; B, [P+T+R vs. P+R]; C, [P+HA+R vs. P+R]. [T+P vs. P]: Plants treated with Trichoderma compared to control plants (water); [P+T+R vs. P+R]: Infected plants treated with Trichoderma compared to untreated infected plants; [P+HA+R vs. P+R]: infected plants treated with HA compared to untreated Infected plants*.

### Plant–*Trichoderma harzianum* M10-*Rhizoctonia solani* interaction

**Figure 4:**
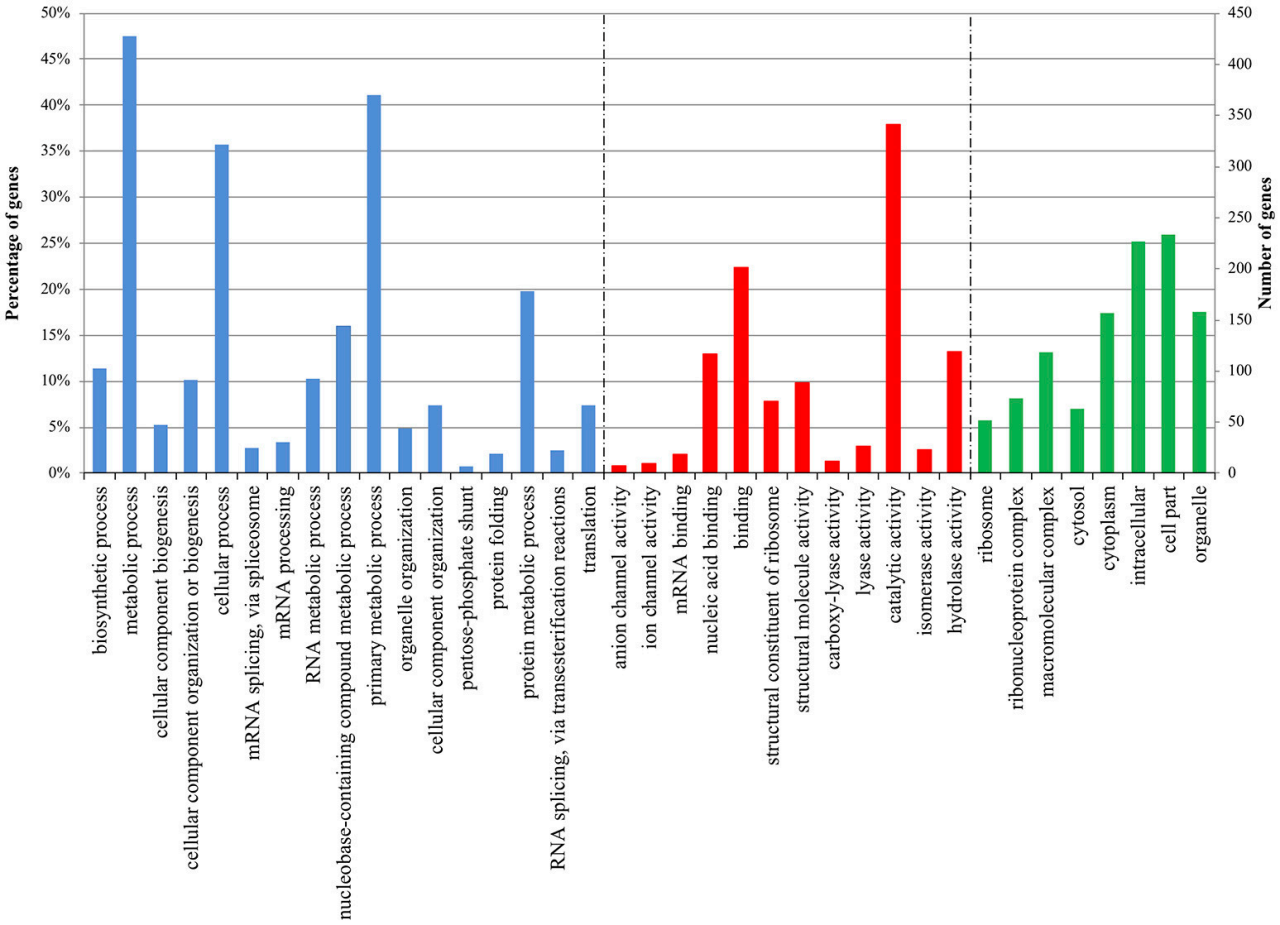
Enriched Gene Ontology (GO) terms distribution of tomato DEGs in plant-*Trichoderma-Rhizoctonia* [P+T+R vs. P+R] interaction 30 days post-inoculum. The GO terms displayed along the X-axis are grouped in three GO categories: Biological Process (blue); Molecular Function (red); and Cellular Component (green). The values on the Y-axis indicate tomato genes in each GO term represented as: on the left, the percentage of total genes; and on the right, the number of genes detected in each GO term.

Results from expression analysis of tomato responses in the plant–*T. harzianum* M10–*R. solani* interaction were compared to those from the plant-pathogen interaction [P+T+R vs. P+R]. A total of 1,142 DEGs were noted, of which 630 genes were over-expressed and 512 were down-regulated and found distributed in the list of pathways identified by Mapman in the “Cellular Function” category (Tables 2, 3B). Among these, the most represented DEGs were involved in “protein synthesis/degradation” (241 genes), “RNA metabolism” (142 genes) and “signaling” (49 genes). Concerning response to biotic stress, 298 genes belonging to several MapMan categories were found to have their expression level modified, with 32 of them also involved in responses to abiotic stress (Figure 2B). Additionally, the occurrence of DEGs associated to JA and SA biosynthesis, cell wall synthesis (27 genes), proteolysis (88 genes), signaling (49 genes), and ethylene (14 genes) was observed (Figure 2B). Remarkably, one of the cellular processes most affected by the tripartite interaction appeared to be the metabolism of oxygenic compounds with 23 genes up-regulated (Figure 2B, Redox state, Peroxidases, Gluthatione-S-transferase). Many genes related to different transcription factors (TFs) were found down-regulated (WRKY, MYB) while bZIP and *DOF* were up-regulated (Figure 2B). Infected plants treated with M10 showed the over-expression of nine genes involved in plant secondary metabolism. Specifically, four genes were associated to phenylpropanoid metabolism, three were involved in isoprenoid biosynthesis, while two genes were associated to flavonoid production (Figure 2B). The GO analysis revealed 37 enriched GO terms, 17 associated to Biological process, 12 to Molecular function and eight to Cellular component, respectively (Figure 4). Most of the enriched GO terms found in the bipartite interaction were in common with those found in the tripartite interaction GO enrichment. However, the number of DEGs identified in the latter analysis was much higher than that obtained for the bipartite interaction (Table 2).

### Plant–HA-*Rhizoctonia solani* interaction

The expression analysis of plant response in the Plant–HA–*R. solani* interaction [P+HA+R vs. P+R] revealed a total of 2,317 DEGs, of which the majority (1,456) were over-expressed (Table 2). In all cases, there were more DEGs activated by the treatment with HA and the pathogen than by the treatments of *Trichoderma* alone or by *Trichoderma* plus *Rhizoctonia*, suggesting a strong plant response to the fungal metabolite. As reported in Table 3C, the most represented MapMan categories were “protein synthesis/degradation” (505 genes), “RNA processing-regulation of transcription” (284 genes), “signaling” (122 genes) and “hormone metabolism” (63 genes).

The response to biotic stress affected the expression of 648 genes, six of which were directly involved in pathogen recognition and plant defense (Figure 2C). Up-regulation of genes involved in ethylene (29 genes), JA (7 genes), and oxygenic compounds (42 genes) pathways was also observed. All transcription factor families putatively involved in defense responses were also represented (ERF, bZIP, WRKY, MYB, DOF) among the up-regulated genes. Furthermore, infected plants treated with HA showed a remarkable activation of secondary metabolism with the over-expression of 44 genes involved in the biosynthesis of flavonoids, isoprenoids, phenylpropanoids and aromatic amino acids.

**Figure 5:**
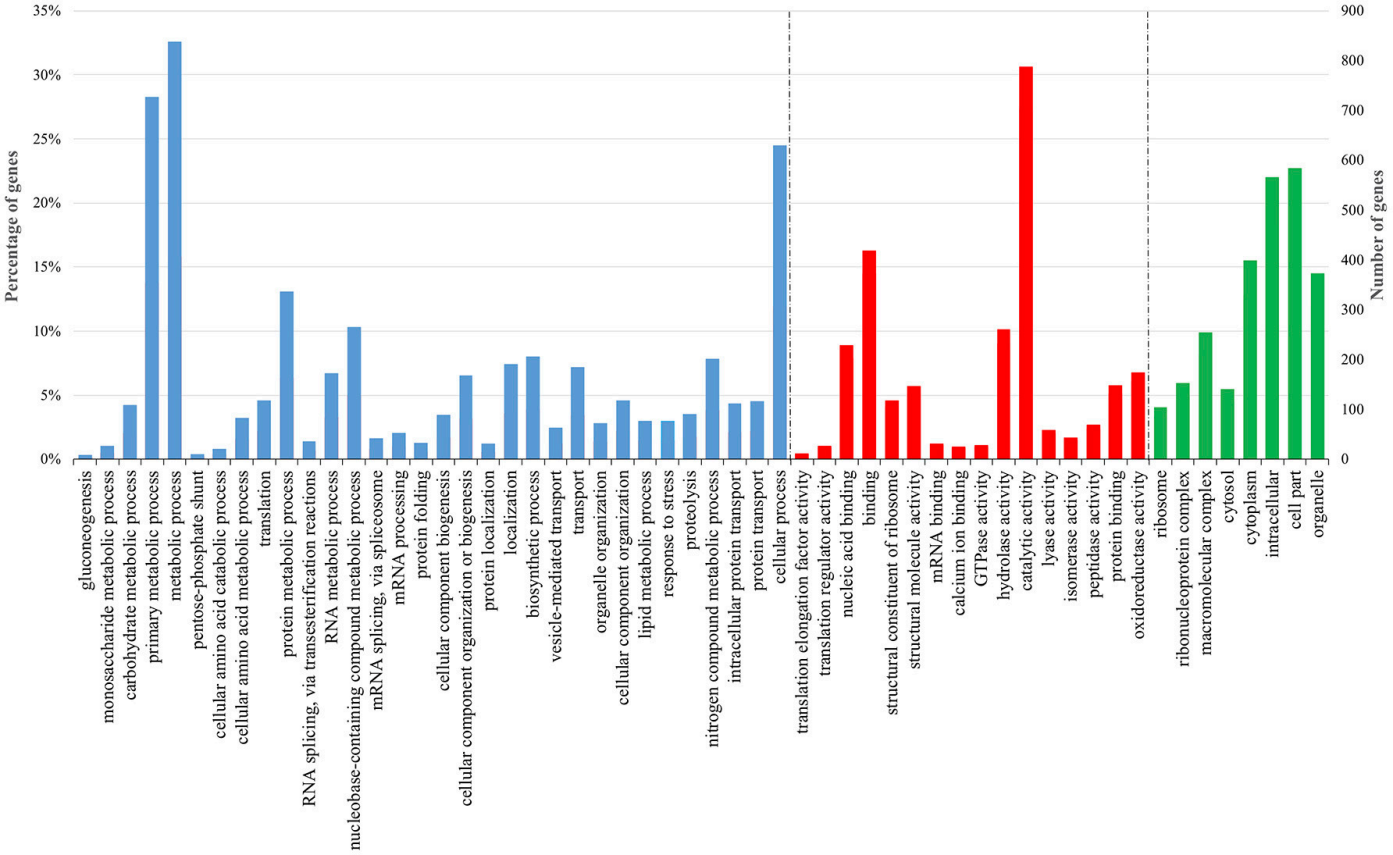
Enriched Gene Ontology (GO) terms distribution of tomato DEGs in plant—harzianic acid—*Rhizoctonia* [P+HA+R vs. P+R] interaction 30 days post-inoculum. The GO terms displayed along the X-axis are grouped in three GO categories: Biological Process (blue); Molecular Function (red); and Cellular Component (green). The values on the Y-axis indicate tomato genes in each GO term represented as: on the left, the percentage of total genes; and on the right, the number of genes detected in each GO term.

GO analysis highlighted the presence of 32 enriched terms belonging to Biological process category, 16 to Molecular function and 8 to Cellular component (Figure 5). The most represented GO terms were “cellular process,” “metabolic process,” and “primary metabolic process” and many genes involved in protein metabolism, transport, localization and response to stress were identified.

### Plant responses comparisons—with and without pathogen

**Figure 6:**
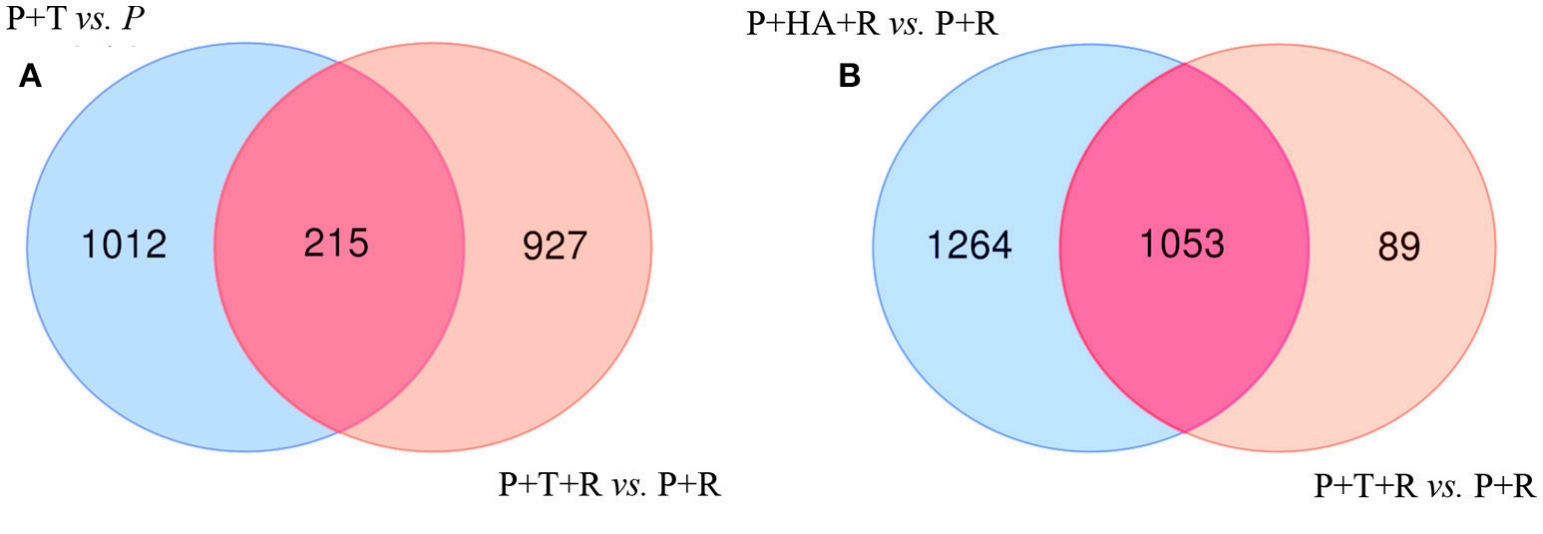
Venn Diagrams showing the number of unique and overlapping DEGs in bipartite and tripartite interactions. **(A)** [P+T vs. P] (non- infected plants treated with M10) vs. [P+T+R vs. P+R] (infected plant treated with M10); **(B)** [P+HA+R vs. P+R] (infected plant treated with HA secondary metabolite) vs. [P+T+R vs. P+R] (infected plant treated with M10).

**Figure 7:**
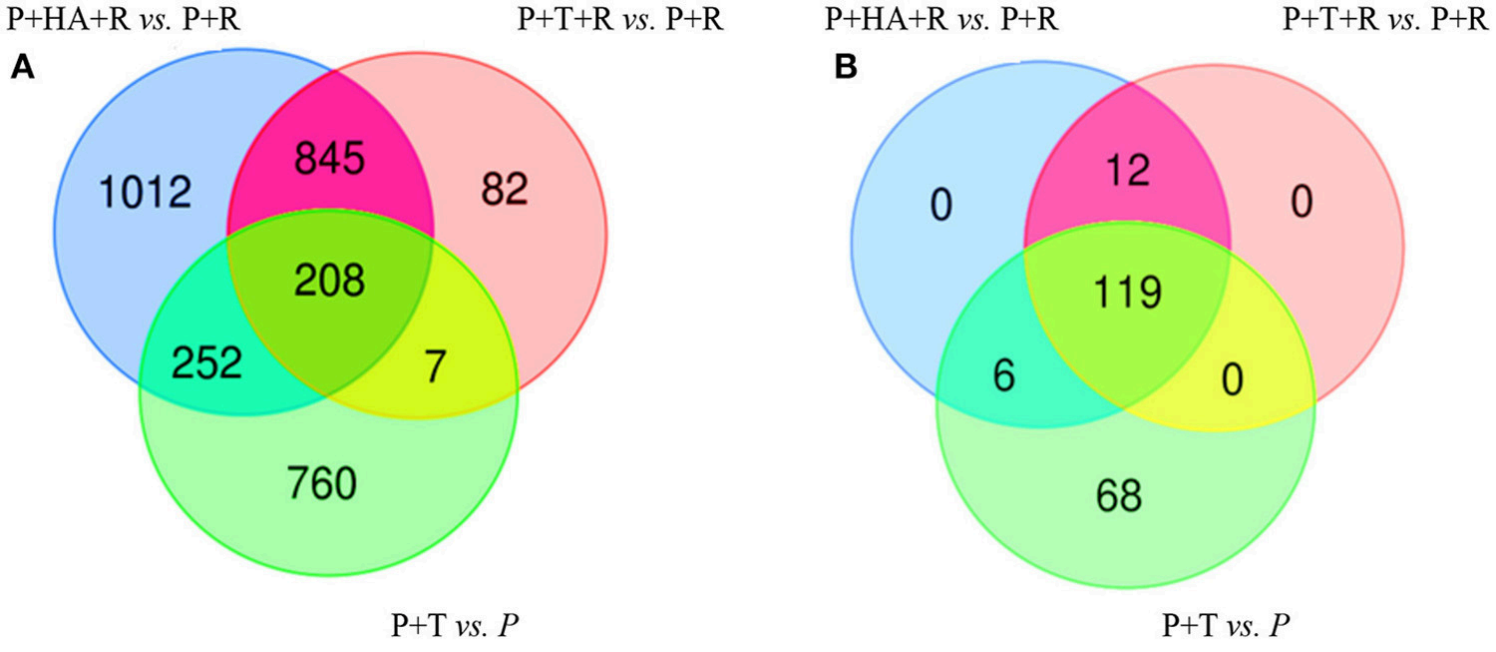
Venn Diagrams showing the number of unique and overlapping DEGs in bipartite and tripartite interactions. **(A)** Comparative analysis of DEGs obtained from [P+T vs. P] vs. [P+T+R vs. P+R] vs. [P+HA+R vs. P+R]; **(B)** Fold changes value of the 208 DEGs found in common in **(A)**; 119 genes resulted over-expressed in all the conditions, 12 were up-regulated in [P+T+R vs. P+R] and [P+HA+R vs. P+R] and down in [P+T vs. P], while 6 genes resulted up-regulated in [P+T vs. P] and [P+HA+R vs. P+R] and down-regulated in [P+T+R vs. P+R]. [P+T vs. P]: plant treated with *Trichoderma* compared to control plants (water); [P+T+R vs. P+R]: infected plants treated with *Trichoderma* compared to infected plants (*R. solani*); [P+HA+R vs. P+R]: infected plants treated with HA compared to infected plants (*R. solani*).

DEGs obtained comparing the expression analysis of the tripartite and bipartite interactions were studied in order to identify common genes mainly associated to *Trichoderma* colonization in plants infected and non-infected by the pathogen [P+T+R vs. P+T]. Interestingly, 215 DEGs were present in both conditions and a significant portion of them (39%) showed opposite expression values (Figures 6A; Table S2). In the comparison analysis of the DEGs from [P+T+R vs. P+R] and [P+HA+R vs. P+R], used to identify the HA-dependent effect in *Rhizoctonia* infected plants, a total of 1,053 genes were found to be shared, while 1,264 and 89 genes resulted specifically associated to [P+HA+R vs. P+R] and [P+T+R vs. P+R], respectively (Figure 6B). The comparison between all interactions ([P+HA+R vs. P+R] vs. [P+T+R vs. P+R] vs. [P+T vs. P]) allowed to identify 208 shared DEGs (Figure 7A). Interestingly, 119 of them were over-expressed in all the conditions, 12 were up-regulated only in infected plants [P+T+R vs. P+R] and [P+HA+R vs. P+R] and down regulated in non-infected plants [P+T vs. P], while 6 genes resulted up-regulated in [P+T vs. P] and [P+HA+R vs. P+R] and down-regulated in [P+T+R vs. P+R] (Figure 7B). Among the up-regulated genes in pathogen-infected plants treated with the beneficial fungus *Trichoderma* or its metabolite HA, many were found to be involved in processes like photosynthesis, cell wall modifications, cell organization and development (Table S3). On the other hand, DEGs common to [P+T vs. P] and [P+HA+R vs. P+R] conditions were mostly associated to photosynthesis, cellular redox state, protein synthesis/degradation and ABC transporters. Finally, 68 genes were up-regulated only in [P+T vs. P] and down-regulated in the multiple interaction ([P+HA+R vs. P+R] and [P+T+R vs. P+R]). Several of these DEGs resulted involved in transcriptional regulation, protein synthesis/degradation, signaling, and hormone metabolism.

### Microarray validation by RT-qPCR

**Figure 8:**
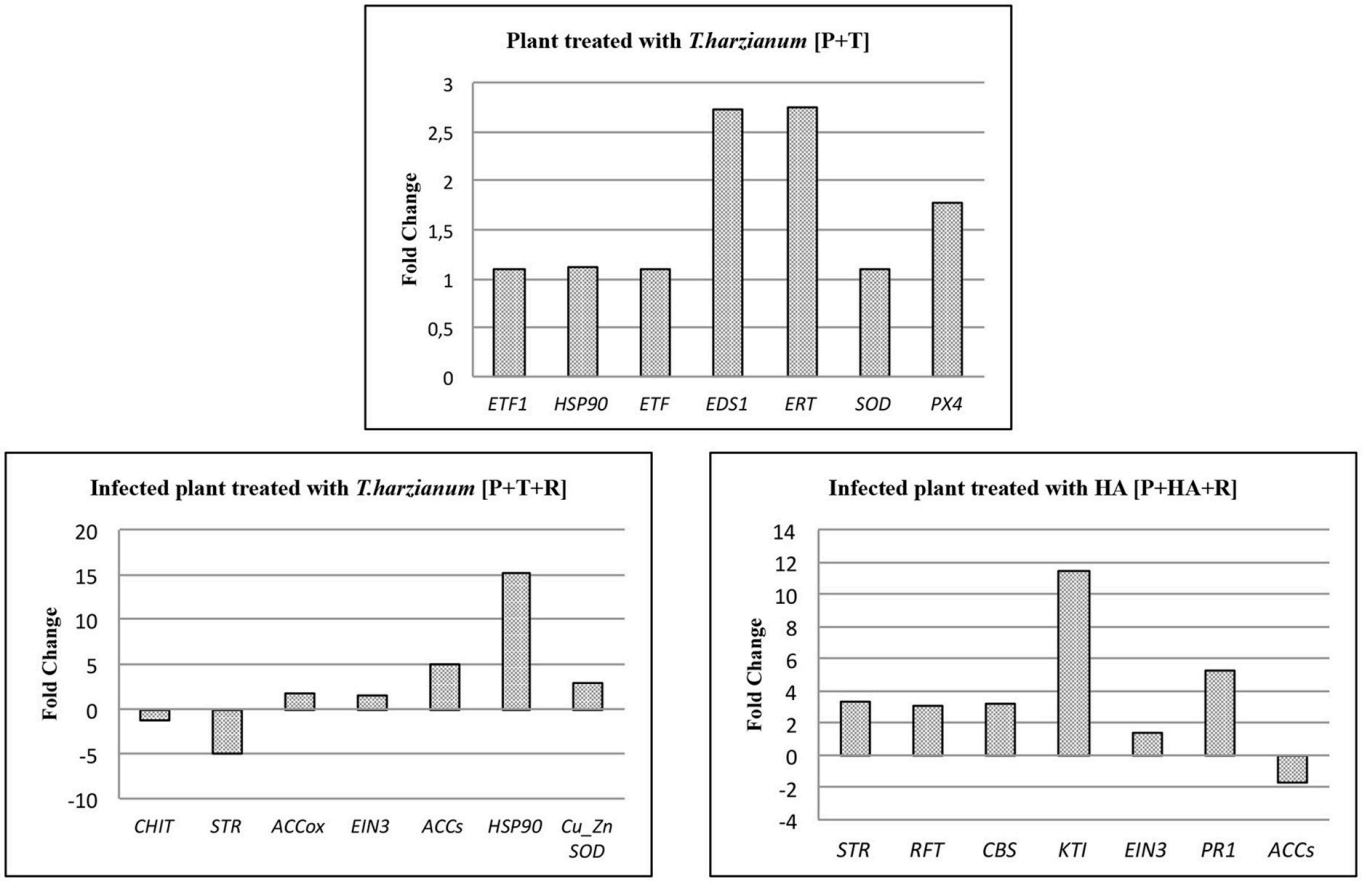
qPCR assay of 7 target genes in each treatment identified by microarray analysis. Bars indicate relative expression measurements (Fold change) of target genes in treated plants respect to the calibrator (control plants). *ETF1*, Ethylene responsive transcription factor 1*; HSP90*, Heat shock protein 90; *ETF*, Ethylene responsive transcription factor*; EDS1*, Enhanced disease susceptibility 1; *ERT*, Ethylene responsive transcript; *SOD*, Superoxide dismutase; *PX4*, Peroxidase 4; *Chi*t, Chitinase; *STR*, Strictosidine synthase family protein; *ACCox*, 1-aminocyclopropane-1-carboxylate oxidase; *EIN3*, Ethylene insensitive 3 class transcription factor; *ACCs*, 1-aminocyclopropane-1-carboxylate synthase; *CuZnSOD*, Cu/Zn-superoxide dismutase copper chaperone; *RFP*, Reticulon family protein; *CBS*, CBS domain-containing protein-like*; KTI*, Kunitz trypsin inhibitor; *PR1*, Pathogenesis related protein 1. The significance of the 2^−ΔΔCt^ values was calculated as *p* < 0.05; Student's *t*-test.

Quantitative real time PCR (qPCR) was used as a validation tool to confirm expression data obtained with microarray analysis. In particular, 21 genes (7 for each interaction) involved in plant defense responses were selected among the DEG lists. As shown in Figure 8, qPCR confirmed the results obtained for DEGs with microarray analysis in each interactions. In particular, in plant-*Trichoderma* interaction, genes involved in ethylene pathway (*ERT* and *EDS1*) and in reactive oxygen species (ROS) detoxification (superoxide dismutase-*SOD*) were found to be significantly up-regulated. In infected plants treated with M10 [P+T+R], *HSP90* as well as 1-aminocyclopropane-1-carboxylate synthase (*ACCs*) were up-regulated, while the expression of both chitinase and strictosidine synthase (*STR*) genes were both down-regulated. In the P+HA+R interaction, the *KTI* (Kunitz trypsin inhibitor) and the *PR1* genes were over-expressed. The results confirmed the reliability of the microarray approach.

### Metabolomic analysis

**Figure 9:**
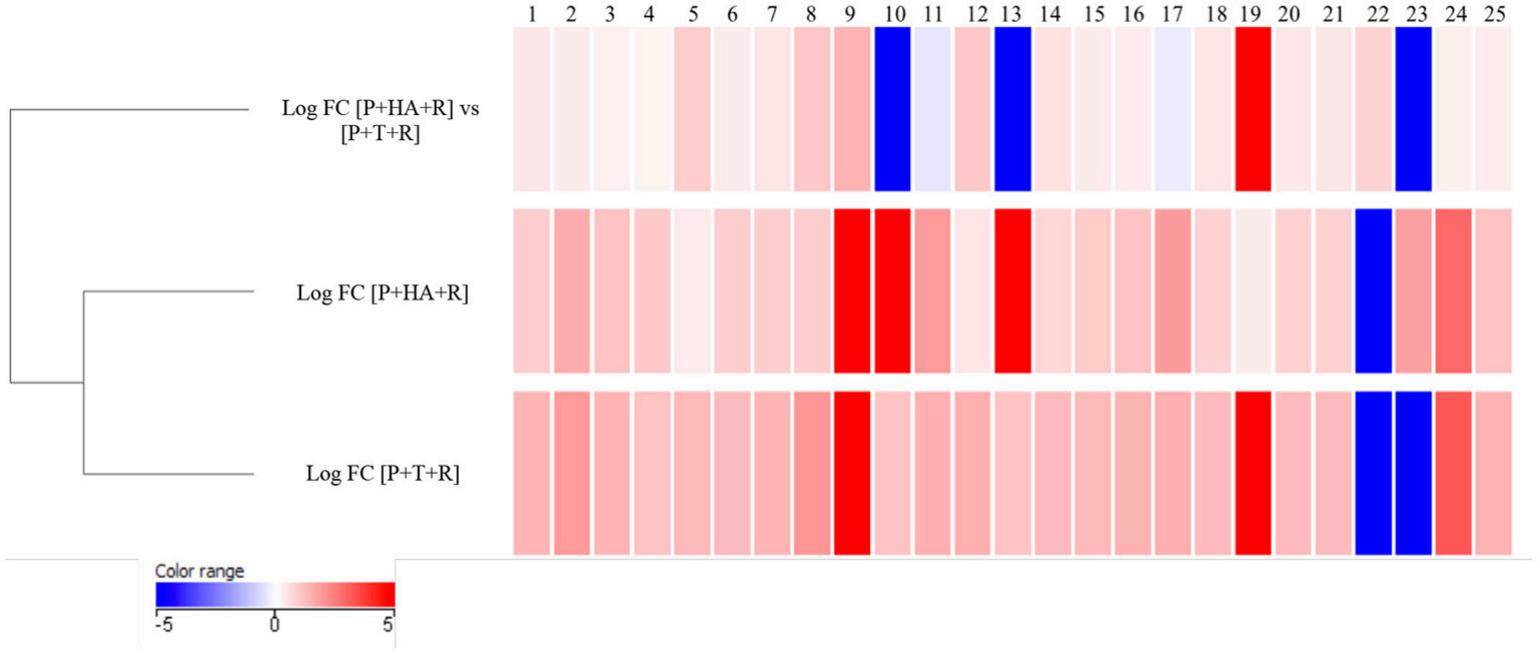
Hierarchical clustering of compounds identified in methanol extract of treated plants using LC-MS in positive mode. Values are expressed in Log FC (normalized by quantile). Each compound was associated to an ID number. The identification of compounds is reported in Table 4.

**Figure 10:**
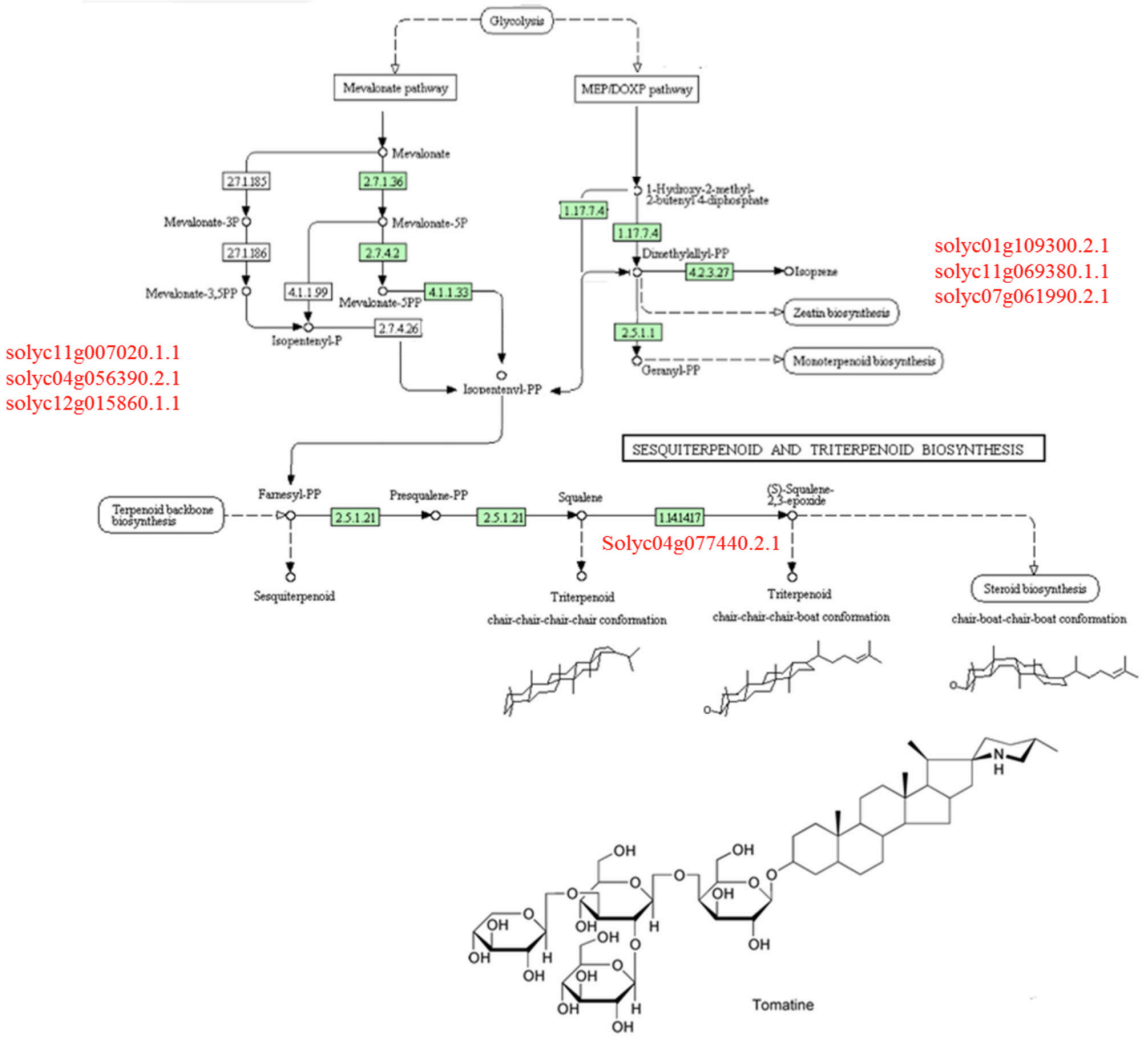
Triterpenoids KEGG biosynthetic pathway involved in the production of steroidal glycoalkaloids. In red are reported key-genes involved in mevalonate (MVA) pathway (on the Left side) and in methyl-erythritol (MEP) pathway (on the Right side).

A comparison of the plant metabolites obtained from the treatments of [P+HA], [P+M10], [P+R] with the water control indicated 10 compounds differentially produced. However, the subsequent bioinformatics analysis did not reveal any molecule annotation predictions, and since it was not possible to determine the molecular formula for many of the compounds, these treatments were not furtherly considered in the comparison analyses (data not shown).The untargeted metabolomic analysis of the methanol extracts from *Rhizoctonia*-infected and M10- or HA-treated plants allowed to identify 25 compounds of which 15 were automatically annotated, while the remaining 10 resulted “unknown” (Table 4). Tomato treated with HA or M10 increased the abundance of more than 90% of the metabolites in comparison with plants only infected by the pathogen, as depicted by the hierarchical clustering shown in Figure 9. Intriguingly, only compound 22 was found to be more abundant in infected plants in comparison with treated groups. Compound 23 was the only substance to be up-regulated in HA-treated plants in comparison to M10-treated plants, whereas in the latter case it was decreased (Figure 9; Table 4). On the other hand, no significant differences in metabolite abundance were found comparing treated groups (i.e., [P+HA+R] vs. [P+T+R]). In fact, out of 25 compounds, 18 showed an FC value close to zero. Interestingly, several putatively identified compounds belong to the class of steroidal glycoalkaloids (SGAs). These molecules are synthetized from isopenthyl-phosphate via mevalonate (MVA) and 2-C-methyl-D-erythritol 4-phosphate (MEP) pathways. In [P+T+R] only genes involved in MVA pathway resulted up-regulated, whereas in [P+HA+R], besides those of the MVA pathway, also three genes involved in the MEP pathway were found over-expressed (Figure 10).

**Table 4:** List of identified compounds in metabolomic analysis.

**Compound**	**[P+T+R] vs. [P+HA+R]**	**[P+HA+R]**	**[P+T+R]**	**Mass**	**RT**	**Molecular formula**	**Putative identification/formula**	**Id. score**	**Composite spectrum**
1	0,444101	0,939036	1,383138	1033,5474	6,9830	C50H83NO21	Tomatine	96.94	1034.5547- 1035,5583-1036.5615
2	0,368933	1,582161	1,951094	577,3991	6,9840	C34H51N5O3	Unknown	97.86	578.4063-579.4097-580.41205
3	0,277088	1,130523	1,407611	1031,5320	6,9000	C50H81NO21	Dehydrotomatine	89.22	1032.5393-1033.5428-1034.5494
4	0,187634	0,995527	1,183162	415,3459	6,9830	C27H45NO2	Tomatidine	98.62	416.35303-417.3561-418.35898
5	0,953433	0,394625	1,348058	575,3835	6,9030	C34H49N5O3	Unknown	NI	576.3907-577.39386
6	0,367650	0,944839	1,312489	103,1002	1,6770	C5H13NO	2-Amino-3-methyl-1-butanol	86.8	104.1074-105.11036
7	0,487186	0,896811	1,383997	137,0480	1,7850	C7H7NO2	Methyl nicotinate-(Trigonelline)	86.76	138.05537-139.05818
8	1,07620330	0,94239616	2,01859950	1048,5581	6,9010	C37H72N22O14	Unknown	NI	525.2868-525.78828-526.2919
9	1,45554260	17,51499600	18,97054000	413,3296	6,9040	C27H43NO2	5alpha-Tomatidan-3-one	81.25	414.33713-415.34024
10	−16,15150800	17,28217900	1,13067050	739,4512	6,9800	C39H65NO12	Lycoperoside D-γ tomatine	67.78	740.4583-741.4614
11	−0,47575950	1,95826770	1,48250820	921,0026	1,4960	NA	Unknown	NI	922.0098-923.0124
12	1,05304240	0,47741318	1,53045560	1063,5562	6,9370	C49H73N15O12	Unknown	NI	1064.5641-1065.5673-1066.5693
13	−13,95523300	1,50859030	1,13067050	380,2901	17,0060	C21H42O4	MG(0:0/18:0/0:0)	76.76	381.29742-382.30084
14	0,55803300	0,76031590	1,31834890	109,0013	1,5240	C4HN2O2	Unknown	NI	110.00861-111.0102
15	0,36856365	0,97294044	1,34150410	162,0528	6,9830	C6H10O5	D-Fructofuranose 1,2′:2,3′-dianhydride	47.48	325.11313-163.0601)
16	0,32262707	1,15023610	1,47286320	386,1730	10,5240	C15H26N6O4S	CysHysLys	62.48	387.18027-388.18338-425.136-409.16208
17	−0,34325027	1,89385750	1,55060720	577,3975	6,5100	C17H45N20O3	Unknown	NI	578.40515-579.4085
18	0,49116898	0,79987430	1,29104330	456,1478	6,9810	C17H28O14	Unknown	NI	457.15494-458.15833
19	17,57123200	0,33272552	17,90395700	731,4610	9,6850	C41H65NO10	Unknown	68.73	732.4684-733.4719
20	0,44027900	0,86345770	1,30373670	1033,5444	6,5100	C50H83NO21	Tomatine	87.43	1034.5525-1035.5558-1036.5592
21	0,45742893	0,86361220	1,32104110	1031,5314	6,9000	C50H81NO21	Dehydrotomatine	58.68	1032.5393-1033.5428
22	0,79794500	−17,17997700	−16,38203000	1050,5719	6,5100	C27H44NO7P	unknown	NI	526.2937-526.7954
23	−15,39738200	1,82587770	−13,57150500	1031,5294	6,2150	C50H81NO21	Dehydrotomatine	60.32	1032.5375-1033.54
24	0,29756737	2,93896630	3,23653360	426,2018	13,3600	C20H30N2O8	Dolychil phosphate D mannose	83.47	427.20892-428.2124
25	0,30474758	1,14425370	1,44900130	763,5235	17,7890	NA	Unknown		764.5308-765.5342

*Compounds are reported with relative normalized logFC values, masses, retention times (RT), molecular formulas, putative identifications, mass scores, and composite spectra. See Figure 9*.

## Discussion

In the case of *Trichoderma*, the establishment of a plant-fungus interaction in most cases produced multiple positive effects (Segarra et al., 2007; Fontenelle et al., 2011; Perazzolli et al., 2011; Brotman et al., 2012; Yoshioka et al., 2012; Martínez-Medina et al., 2017; Nawrocka et al., 2018). The objective of this work was to use transcriptomic and metabolomic analysis to obtain new insights into the molecular and biochemical processes underlying tomato resistance induced by the plant beneficial fungus *T. harzianum* M10 or its metabolite HA, both in healthy and pathogen-infected plants.

### Plant responses induced by *Trichoderma* colonization

In the bipartite interaction *Trichoderma*-plant [P+T vs. P], many genes involved in defense responses as well as in growth and development were found differentially expressed. Germin-like proteins and oxalate oxidase germin-like enzymes were over-represented indicating the activation of hydrogen peroxide production and defense responses against fungal pathogens (Wang et al., 2013). The accumulation of oxalate oxidase germin-like enzymes in the absence of the pathogen ([P+T vs. P]) may be caused by *Trichoderma* root colonization (Martínez-Medina et al., 2017). The presence of *Trichoderma* activated several hormone-signaling pathways (Contreras-Cornejo et al., 2015). For example, many genes directly involved in ethylene signaling, like ethylene receptors (*ER*), ethylene-responsive transcription factors (*ERF*s) and 1-aminocyclopropane-1-carboxylic acid synthase (*ACC*s) were found up-regulated. Microbial hydrolysis of ACC released in root exudates helps to maintain low ethylene levels and, at the same time, increases nitrogen bioavailability in soil, thus favoring plant growth (Glick et al., 2007; Viterbo et al., 2010).

### *Trichoderma* primes plant defense responses

The genes encoding cathepsin B and epoxide hydrolase were over-expressed, suggesting that the induction of programmed cell death (PCD) by *Trichoderma* could be related to endoplasmic reticulum (ER) stress (Cai et al., 2018) in [P+T+R vs. P+R]. Epoxide hydrolase is essential to detoxify ROS, and it also seems to be involved in cutin biosynthesis, the main cuticle component that acts as a physical barrier against pathogen penetration (Pinot et al., 1992), and is involved in the process of biosynthesis as a precursor of JA (Hamberg and Gardner, 1992; Ziegler et al., 1997). Furthermore, the over-expression of two ERFs and ethylene insensitive 3 transcription factor (EIN3), which act as regulators of the plant defense response (Alonso et al., 2003; Yamazaki and Hirose, 2003; Zhong et al., 2009; An et al., 2010), confirmed the involvement of JA and ethylene signaling pathways in the establishment of ISR by *Trichoderma*. This is in accordance with the production of ROS, the accumulation of phenylalanine ammonia-lyase (PAL) and cinnamyl-alcohol dehydrogenase (CAD) transcripts and the synthesis of phenylpropanoids, metabolites that are produced during response to pathogen attack (Korkina, 2007). In general, these results demonstrate that pre-colonization by *Trichoderma* induces a stronger reaction of the plant upon challenge by a pathogen as compared to untreated plants (Lorito et al., 2010; Mauch-Mani et al., 2017).

### Tradeoffs between growth and defense

The comparison between the interactions [P+T+R vs. P+R] and [P+T vs. P] identified 215 shared genes, 83 of which showed opposite expression values (Figure 6A; Table S2). Many of the latter group, such as those encoding for NAC domain protein and transcription factors, cytochrome P450, subtilisin-like protease etc., were over-expressed in the absence but down-regulated in the presence of the pathogen. The lower expression of such genes may be due to the activation of defense responses to the pathogen with a consequent inhibition of other processes (development processes), in order to balance the energy framework. In any case, our findings only refer to an early phase (48 HPI) of the studied interaction, thus we cannot exclude that later on *Trichoderma* may promote growth also in the presence of the pathogen. Significantly changed pathway analysis indicated a strong involvement of the energy metabolism to support a simultaneous activation of growth and defense related processes (Harman et al., 2004b; Segarra et al., 2007; Shoresh and Harman, 2008). In fact, *Trichoderma* up-regulated plant genes involved in TCA, glycolysis, photosynthesis, and gluconeogenesis, as well as ethylene biosynthesis. Interestingly, we found several defense related genes whose transcription was enhanced by *Trichoderma* compared to the *Rhizoctonia*-treatment only.

### HA boosts the plant immune response

The transcriptomic analysis of [P+HA] samples did not reveal any DEGs. However, the absence of a plant response to this treatment is probably not due to a lack of effect caused by HA, since other studies using the same *Trichoderma* compound demonstrated a clear effect also on tomato (Vinale et al., 2013, 2014). Most likely, the timing of collection of the plant material for the RNA extraction used in this work was not appropriate. A recent study by Stringlis et al. (2018) demonstrated that the *Arabidopsis* transcriptional response to treatments with a beneficial strain of *Pseudomonas* or two different flagellins plus one chitin preparation had different kinetics when purified substances were compared to the living microbes. In particular, chitin triggers the strongest activation of plant responses and the highest number of DEGs within 0.5 h post-inoculation (HPI), after which the transcriptional response gradually diminished over time. Furthermore, the effect of HA, also known to be a siderophore (Vinale et al., 2013), could be dependent upon the nutritional status of the plants. Trapet et al. (2016) demonstrated that microbial siderophores affect plant gene expression mainly on iron-deficiency conditions, and this was not the case in our study. On the other hand, it can be speculated that HA has a priming effect on gene expression since the number of DEGs identified in the [P+HA+R vs. P+R] was greater than those observed in the interactions with the *Trichodema* fungus [P+T+R vs. P+R], and 60% of those DEGS were over-expressed. Furthermore, the establishment of a priming status may have allowed HA to perform as well as the living fungus in the biocontrol experiment.

In the comparison of [P+T+R vs. P+R] with [P+HA+R vs. P+R] datasets, the plant response to HA-treatment resulted more intense in terms of activation of stress-induced and resistance genes. Transcripts of heat shock (*HSP*), early-response to dehydration and universal stress proteins, as well as of molecular chaperones (DNAK, DNAJ, DNAJ2), were also increased. In addition, overexpression of osmotin-like genes, involved in biotic and abiotic stress response was also observed (Hanin et al., 2011; Hakim et al., 2017).

The simultaneous over-expression of HSP90 and co-molecular chaperones is reported to be linked to the accumulation of disease resistance (R) genes encoding pathogen receptors (Holt et al., 2005). This is in agreement with the overexpression of genes encoding resistance proteins with an N-terminal coiled-coil (CC) domain, a central nucleotide-binding site (NBS) domain and a C-terminal leucine-rich repeat (LRR) domain (CC-NBS-LRR) found in the presence of HA and with the activation of SA, JA and ethylene biosynthetic pathway. The whole activation of defense responses was indicated by the over-expression of genes coding for pathogenesis-related proteins (PR), such as PR1, oxalate oxidase-like germin 171 and kunitz-type proteinase inhibitor. The up-regulation of *PR1* could be related to the activation of systemic acquired resistance (SAR) as well as to an ISR status. Interestingly, we found a simultaneously expression of genes involved in SA and JA biosynthesis only when plants were treated with HA in the presence of *Rhizoctonia* and this may have resulted in an enhanced protection against the pathogen (Verhagen et al., 2006).

### *Trichoderma* and HA increase plant chemical weapons

The untargeted metabolomic analysis revealed the presence of 25 compounds mainly annotated in the SGAs class. SGAs are specialized anti-nutritional metabolites constitutively produced in plants and frequently reported as determinants of resistance to fungal attack (Friedman, 2002; Bednarek and Osbourn, 2009).

In tomato, α-tomatine is important in the defense against fungi because of its specific effect on sterols that leads to membrane leakage (Sandrock and VanEtten, 1998). Lycoperoside belongs to the same chemical class and, similarly to other SGAs, its biosynthesis is strictly dependent on cholesterol. The level of lycoperoside was significantly increased in [P+HA+R], which is in agreement with the activation, as found in the microarray analysis, of cholesterol biosynthesis observed in pathogen-infected plants treated with HA. On the other hand, compounds such as tomatidanol and tomatine were predominant in plants infected by *R. solani* and treated with M10. These results indicate an activation of terpenoid and polyketide pathways with the biosynthesis of alkaloids related to chemical defense. Furthermore, N-methyl-nicotinic acid (Trigonelline), another important SM involved in defense response (Kraska and Schönbeck, 1993) was found particularly abundant in infected plants colonized by *Trichoderma*, which suggest a positive correlation between the presence of the biocontrol fungus and the accumulation of this compound.

## Conclusions

A microarray analysis was used to study tomato gene expression in the interaction with the fungal biocontrol agent *T. harzianum* M10 or its secondary metabolite HA, in the presence of the soil-borne root pathogen *R. solani*. The over-expression of genes involved in glycolysis, TCA and photosynthesis, as well as those implicated in cell wall remodeling, is related to the growth promotion effect of *T. harzianum* M10 on healthy plants. In addition, despite the initial activation of defense responses, the promoted expression of genes involved in cellular homeostasis is in agreement with the beneficial nature of the *Trichoderma-*plant interaction (Hermosa et al., 2013).

**Figure 11:**
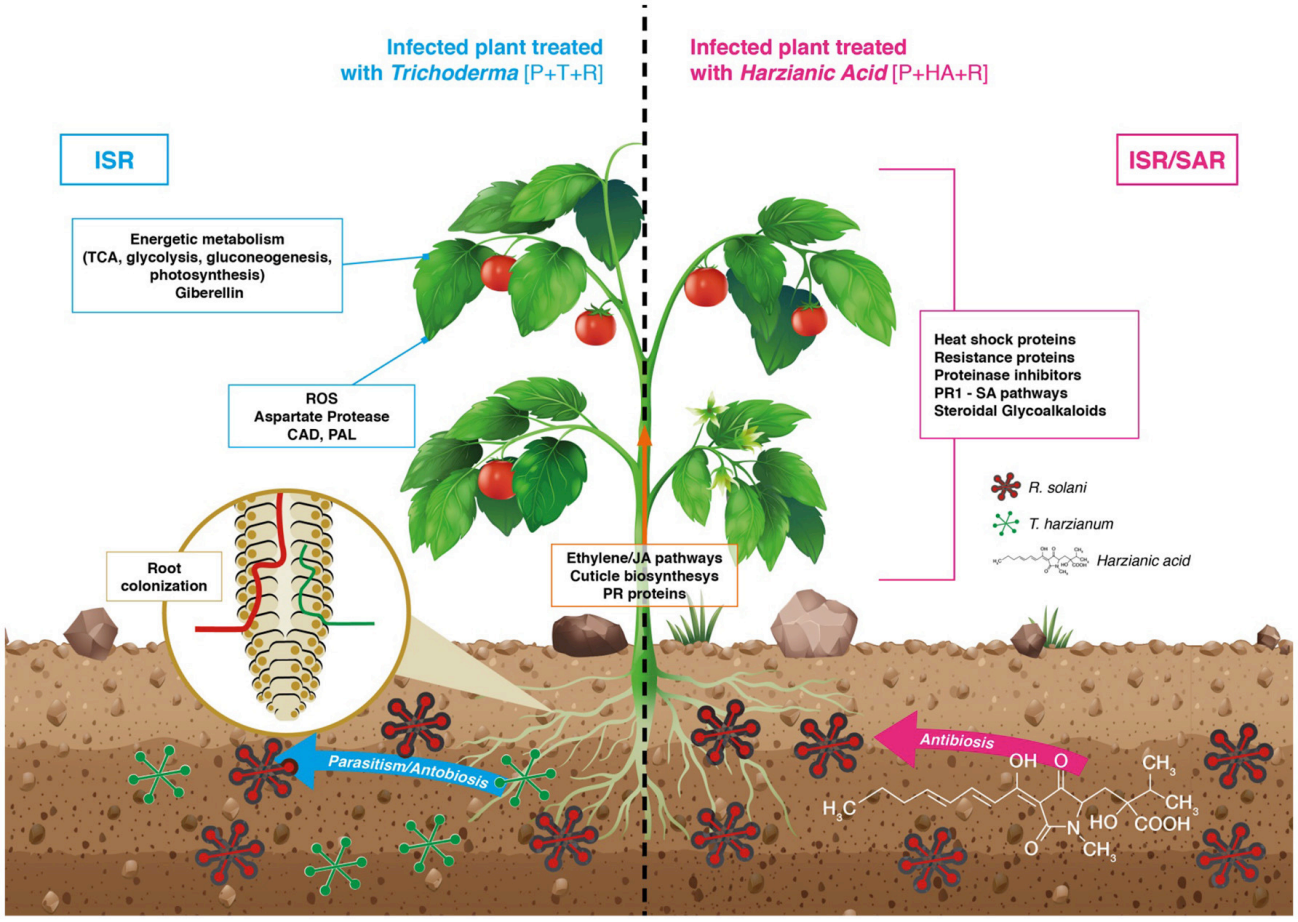
Model of response of tomato plant infected with the soil pathogen *Rhizoctonia solani* (see figure legend) when pre-treated with *Trichoderma harzianum* or its bioactive metabolite harzianic acid (HA). In the soil *T. harzianum* limits pathogen proliferation through its mycoparasitic action and the release of antibiotic compounds like Harzianic Acid (HA). In the presence of pathogens, *T. harzianum* and its metabolite HA elicit defense responses mainly by the production of toxic secondary metabolites, strengthening of physical barriers and triggering ethylene/jasmonic acid biosynthetic pathways (induced systemic resistance-ISR). Additionally, HA carries out its beneficial effect activating salicylic acid (SA) pathway (systemic acquired resistance-SAR). Besides pathogen control, *T. harzianum* also enhances plant energetic metabolism thus promoting its growth and development.

The presence of the pathogen resulted in a strong overexpression of genes involved in different resistance mechanisms. When infected plants were colonized by *T. harzianum* or treated with HA, physical and chemical defenses involving cuticle biosynthesis and production of toxic secondary metabolites, were activated. Both treatments stimulated ethylene/JA pathways related to ISR activation. In particular, HA also up-regulated the SA pathway and the SAR marker PR1, thus causing the co-induction of ISR and SAR response (Van Wees et al., 2000; Figure 11).

This work provides a first overall view of the molecular response triggered in plant by the bioactive and commonly produced fungal metabolite HA. *T. harzianum* and HA treatments demonstrated generally comparable changes in defense-related gene expression and efficacy in *R. solani* containment in controlled conditions. Direct use of new, biocontrol-related fungal metabolites may help to overcome problems of efficacy, reliability and persistency of the effect related to the use a living beneficial microorganisms (Vinale et al., 2008).

## Author contributions

GM executed microarray and molecular experiments, processed samples, conducted molecular studies, performed data elaboration and statistical analyses, interpreted the results, and was significantly involved in writing the manuscript. AS processed the microarray experiments, collected and analyzed data, interpreted the results. ME assisted in the experimental design, the microarray analysis, data interpretation. FV defined experimental protocols for the metabolomics analyses, performed data elaboration and analyses. SL, AP, MN, and NL executed the experiments and developed protocols, assisted in sample processing and data collection, and were involved in writing sections of the manuscript. ML assisted in experimental designs, interpretation of results, and writing of the manuscript. SW designed the study experiments and protocols, carried out sampling, coordinated molecular studies and data analysis, interpreted the results, and was significantly involved in writing of the manuscript.

### Conflict of interest statement

The authors declare that the research was conducted in the absence of any commercial or financial relationships that could be construed as a potential conflict of interest. The handling Editor declared a shared affiliation, though no other collaboration, with one of the authors FV.
