# Supplementary material for: Modulation of Tomato Response to Rhizoctonia solani by Trichoderma harzianum and Its Secondary Metabolite Harzianic Acid
Source: Front Microbiol. 2018 Aug 30;9:1966. doi: 10.3389/fmicb.2018.01966 (PMC6127634; doi:10.3389/fmicb.2018.01966)
Supplement: Supplementary file 1 [file Table_1.DOCX]

**Table S1: Genes selected for microarray analysis validation by qPCR.** Treatments: P+T: Plant treated with *Trichoderma*; P+T+R: *R.solani* infected plants treated with *Trichoderma*; P+HA+R: *R.solani* infected plants treated with HA. For each gene is reported the ID (Solyc), annotation and primer sequences.

| Treatments | Gene | ID | Annotation | Primers |
| --- | --- | --- | --- | --- |
|  | *TUB* |  | Alpha-tubulin | Fw: AGCTCATTAGCGGCAAAGAA  Rv:AGTACCCCCACCAACAGCA |
| P+T | *ETF1* | Solyc05g052040.1.1 | Ethylene responsive transcription factor 1a | \| Fw: GGAGATTCGTGACCCAACTAG  Rv: CTCCCTCTGAGCCTAAATGC \| \| --- \| |
| P+T | *HSP90* | Solyc06g036290.2.1 | Heat shock protein 90 | \| Fw: CTCTTTATTCGCCTTGTTCCTG \| \| --- \| \| Rv: CCTGTAGAGCTTCCATGAACTC \| |
| P+T | *ETF* | Solyc08g078190.1.1 | Ethylene responsive transcription factor | \| Fw: TGGTTCTAGAGTTTGGCTTGG \| \| --- \| \| Rv: CGGAGCTTGAATACGACAGAG \| |
| P+T | *EDS1* | Solyc06g071280.2.1 | Enhanced disease susceptibility 1 | \| Fw: AGGGACGAAGAGACTGGATAG \| \| --- \| \| Rv: GCCTAGCCCTGATCAAGTAAG \| |
| P+T | *ERT* | Solyc10g006130.1.1 | Ethylene responsive transcription factor 3a | \| Fw: AGAAAACATCCTAGATCGCCG \| \| --- \| \| Rv: CCCCTTCACAATCACCATCTTC \| |
| P+T | *SOD* | Solyc01g067740.2.1 | Superoxide dismutase | \| Fw: AGATTCAGCAACTAGCGGTG \| \| --- \| \| Rv: CAACCCCAATTCAAAAGGCG \| |
| P+T | *PX4* | Solyc04g071890.2.1 | Peroxidase 4 | \| Fw: TGCTGGCTTACTTCGTCTTC \| \| --- \| \| Rv: TCCATCTAGCAACACTGAACC \| |
| P+T+R | *Chit* | Solyc01g097270.2.1 | Chitinase | \| Fw: CGCCCGAATATTGTTCACCTAG \| \| --- \| \| Rv: ACAGCATTCAAATCCCACCC \| |
| P+T+R | *STR* | Solyc02g082900.2.1 | Strictosidine synthase family protein | \| Fw: TTCTTATCCGACCCATGTGC \| \| --- \| \| Rv: CTACGTCTTCGGGTGCTAATAG \| |
| P+T+R | *ACCox* | Solyc02g036350.2.1 | 1-aminocyclopropane-1-carboxylate oxidase | \| Fw: TGGAGAAACTAGCAGAGCAAC \| \| --- \| \| Rv: GATCAGGCTTAGGACATGGTG \| |
| P+T+R | *EIN3* | Solyc01g009170.2.1 | Ethylene insensitive 3 class transcription factor | \| Fw: TCTTGATTTCTTCCCTGCTCC \| \| --- \| \| Rv: CATCTTATCCCTCCACATCCTC \| |
| P+T+R | *ACCs* | Solyc05g050010.2.1 | 1-aminocyclopropane-1-carboxylate synthase | \| Fw: CCTGGATCTTCGTTCAATTGC \| \| --- \| \| Rv: CAAGTGCGATCTCCATTGTTTG \| |
| P+T+R | *HSP90* | Solyc06g036290.2.1 | Heat shock protein 90 | \| Fw: CTCTTTATTCGCCTTGTTCCTG \| \| --- \| \| Rv: CCTGTAGAGCTTCCATGAACTC \| |
| P+T+R | *CuZn SOD* | Solyc08g079830.2.1 | Cu/Zn-superoxide dismutase copper chaperone | \| Fw: AATTGTACAGCCTACCACTCG \| \| --- \| \| Rv: AATCAGGTCAGCAACTCTCAG \| |
| P+HA+R | *STR* | Solyc02g082900.2.1 | Strictosidine synthase family protein | \| Fw: TTCTTATCCGACCCATGTGC \| \| --- \| \| Rv: CTACGTCTTCGGGTGCTAATAG \| |
| P+HA+R | *RFP* | Solyc01g095200.2.1 | Reticulon family protein | \| Fw: CTCATATCCCAGAAGTCCACC \| \| --- \| \| Rv: CCAGCAATCACAGCAAGAAAC \| |
| P+HA+R | *CBS* | Solyc02g064950.2.1 | Cystathionine beta-synthase | \| Fw: TCTTCCGAAATCCGACCATG \| \| --- \| \| Rv: ACGGAGAGGAAACATTTAGCG \| |
| P+HA+R | *KTI* | Solyc03g098730.1.1 | Kunitz trypsin inhibitor | \| Fw: CCCAAAATGCTCAATCTTCCG \| \| --- \| \| Rv: TGTTGATTGTCCATACCGTCG \| |
| P+HA+R | *EIN3* | Solyc01g009170.2.1 | Ethylene insensitive 3 class transcription factor | \| Fw: TCTTGATTTCTTCCCTGCTCC \| \| --- \| \| Rv: CATCTTATCCCTCCACATCCTC \| |
| P+HA+R | *PR1* | Solyc09g007010.1.1 | Pathogenesis related protein PR-1 | \| Fw: ACTATGCCAACTCAAGAGCG \| \| --- \| \| Rv: GGTTGGTAGCGTAGTTATAGTCTG \| |
| P+HA+R | *ACCs* | Solyc05g050010.2.1 | 1-aminocyclopropane-1-carboxylate synthase | \| Fw: CCTGGATCTTCGTTCAATTGC \| \| --- \| \| Rv: CAAGTGCGATCTCCATTGTTTG \| |
