# Supplementary material for: Modulation of Tomato Response to Rhizoctonia solani by Trichoderma harzianum and Its Secondary Metabolite Harzianic Acid
Source: Front Microbiol. 2018 Aug 30;9:1966. doi: 10.3389/fmicb.2018.01966 (PMC6127634; doi:10.3389/fmicb.2018.01966)
Supplement: Supplementary file 2 [file Table_2.DOCX]

**Table S2: Identification, relative expression values and annotation of 83 shared DEGs with an opposite regulation found in the comparative analysis (P+T+R *vs* P+T).** P+T: Plant treated with *Trichoderma*; P+T+R: *R.solani* infected plants treated with *Trichoderma.*

| **ID** | **P+T *vs* P** | **P+T+R *vs* P+T** | **Annotation ITAG SL 2.40** |
| --- | --- | --- | --- |
| Solyc03g114920.1.1 | 1,523 | -3,103 | Pentatricopeptide repeat-containing protein |
| Solyc08g074560.2.1 | 1,457 | -3,473 | Uncharacterized aarF domain-containing protein kinase 1 |
| Solyc06g065820.2.1 | 2,397 | -4,632 | Ethylene-responsive transcription factor 7 |
| Solyc10g005590.2.1 | 1,417 | -2,839 | Nuclear cap-binding protein subunit 2 |
| Solyc09g014350.2.1 | 2,135 | -2,817 | Glycerol-3-phosphate acyltransferase 6 |
| Solyc11g011040.1.1 | 2,904 | -2,675 | ADP-ribosylation factor |
| Solyc09g089580.2.1 | 2,678 | -3,826 | 1-aminocyclopropane-1-carboxylate oxidase-like protein |
| Solyc10g084400.1.1 | 2,977 | -3,017 | Glutathione S-transferase |
| Solyc06g076050.2.1 | 2,123 | -3,286 | Ankyrin repeat domain-containing protein 28 |
| Solyc07g066330.2.1 | 3,297 | -4,576 | NAC domain protein IPR003441 |
| Solyc09g092390.2.1 | 1,744 | -2,465 | Adenosylhomocysteinase |
| Solyc10g075070.1.1 | -2,023 | 3,849 | Non-specific lipid-transfer protein |
| Solyc06g049050.2.1 | -5,11 | 3,081 | Expansin |
| Solyc10g080720.1.1 | 1,697 | -4,665 | Polypyrimidine tract binding protein |
| Solyc11g017140.1.1 | 1,749 | -2,43 | C2-H2 zinc finger protein |
| Solyc08g081480.2.1 | 2,609 | -4,305 | cytochrome P450 |
| Solyc04g040200.2.1 | 3,515 | -3,797 | retinaldehyde-binding/triple function C-terminal |
| Solyc01g087680.2.1 | 2,705 | -2,461 | Unknown |
| Solyc03g078520.2.1 | 2,15 | -3,415 | Receptor like kinase, RLK |
| Solyc11g062130.1.1 | 2,869 | -2,378 | Mitochondrial ADP/ATP carrier |
| Solyc10g017530.2.1 | 2,209 | -3,199 | ATP-dependent RNA Helicase |
| Solyc10g039280.1.1 | 2,713 | -4,135 | Dual specificity protein phosphorylation protein |
| Solyc03g115110.2.1 | 2,34 | -2,413 | ATP synthase gamma chain |
| Solyc12g042540.1.1 | 2,428 | -2,65 | Unknown Protein |
| Solyc03g082960.2.1 | 2,167 | -2,942 | Serine/threonine phosphatase family protein |
| Solyc08g075700.2.1 | 1,408 | -3,303 | 60S ribosomal protein L13 |
| Solyc06g082280.2.1 | 2,365 | -4,668 | Serine-threonine protein phosphatase |
| Solyc11g044320.1.1 | 3,059 | -3,046 | Single myb histone |
| Solyc02g077330.2.1 | 1,935 | -3,265 | GDSL esterase/lipase At5g45950 |
| Solyc03g019950.2.1 | -2,314 | 3,019 | DNA chromosome 5 P1 clone MAC12 |
| Solyc12g005330.1.1 | -2,914 | 5,365 | 50S ribosomal protein L2 |
| Solyc09g075440.2.1 | 2,913 | -2,564 | Ethylene receptor |
| Solyc09g011480.2.1 | 2,42 | -2,316 | Rop guanine nucleotide exchange factor 1 _ |
| Solyc02g092920.2.1 | 1,507 | -2,403 | Cadmium-transporting ATPase |
| Solyc01g080540.2.1 | 1,482 | -3,116 | Unknown |
| Solyc01g080090.2.1 | 1,452 | -3,874 | Unknown |
| Solyc05g056010.2.1 | 2,649 | -2,841 | phosphatase (Fragment) (AHRD V1 **-- D7U0L7_VITVI) |
| Solyc01g101240.2.1 | -5,56 | 3,424 | Aspartic proteinase |
| Solyc07g064930.2.1 | 2,029 | -3,394 | Protein grpE |
| Solyc06g065440.1.1 | 1,409 | -3,815 | Zinc finger family protein |
| Solyc11g006690.1.1 | 2,02 | -2,581 | 40S ribosomal protein S17-like protein |
| Solyc02g082070.2.1 | 2,784 | -3,818 | Unknown |
| Solyc05g049950.2.1 | 1,769 | -3,803 | nuclear ribonucleoprotein-associated protein B |
| Solyc02g085210.1.1 | 1,758 | -3,386 | UPF0567 protein C13orf39 homolog |
| Solyc04g007160.1.1 | 1,524 | -3,591 | Alpha-glucosidase |
| Solyc07g062140.2.1 | 2,713 | -2,891 | Alpha alpha-trehalose-phosphate synthase |
| Solyc11g011380.1.1 | -2,218 | 6,381 | Glutamine synthetase |
| Solyc02g021760.2.1 | 1,683 | -3,104 | mRNA 3&apos-end-processing protein yth1 |
| Solyc06g007340.2.1 | -1,479 | 2,662 | Gamma-interferon-inducible lysosomalthiol reductase |
| Solyc01g010660.2.1 | 2,064 | -2,997 | Unknown |
| Solyc10g007120.2.1 | 1,848 | -5,168 | DNA chromosome 3 BAC clone F4B12 |
| Solyc03g098240.2.1 | 2,307 | -3,681 | Glutamate decarboxylase |
| Solyc01g107250.2.1 | 1,929 | -3,12 | LRR receptor-like serine/threonine-protein kinase, RLP |
| Solyc01g068150.2.1 | 2,528 | -3,375 | 11 contig 1 DNA sequence |
| Solyc02g083810.2.1 | 2,812 | -2,672 | Ferredoxin--NADP reductase |
| Solyc03g044060.2.1 | 1,433 | -2,565 | Formin 3 |
| Solyc02g037550.2.1 | 1,941 | -3,435 | Unknown |
| Solyc02g092680.1.1 | 1,837 | -4,804 | Subtilisin-like protease |
| Solyc02g092240.2.1 | 1,663 | -2,907 | protein (Fragment) (AHRD V1 *-*- Q0IY85_ORYSJ)" |
| Solyc01g079760.2.1 | 1,457 | -2,806 | Unknown |
| Solyc02g079590.2.1 | 2,274 | -2,892 | Serine/threonine kinase receptor |
| Solyc02g088780.2.1 | 1,811 | -2,654 | Ribosome biogenesis protein ytm1 |
| Solyc10g062180.1.1 | 3,151 | -4,646 | Polyadenylate-binding protein |
| Solyc06g075110.2.1 | 2,564 | -3,775 | ketoglutarate reductase trans-splicing related 1 |
| Solyc03g111730.2.1 | 3,207 | -2,939 | Cathepsin B-like cysteine proteinase |
| Solyc06g071310.2.1 | 2,137 | -2,945 | LIM domain protein |
| Solyc03g116890.2.1 | 2,157 | -3,261 | WRKY transcription factor 2 |
| Solyc06g071470.2.1 | 2,374 | -3,97 | Peroxisomal membrane protein PEX14 |
| Solyc06g008310.2.1 | 1,749 | -2,42 | Elongator complex protein 2 |
| Solyc03g007670.2.1 | 1,998 | -3,345 | SGT1 |
| Solyc01g007340.2.1 | 2,267 | -3,082 | Acetyl-coenzyme A carboxylase carboxyl transferase subunit beta |
| Solyc09g075000.2.1 | 2,093 | -3,12 | repeat protein |
| Solyc02g090970.1.1 | 1,05 | -3,877 | Serine/threonine-protein kinase 24 |
| Solyc01g010870.2.1 | 2,172 | -2,783 | Unknown |
| Solyc07g064940.2.1 | 2,766 | -3,988 | Thioredoxin family protein |
| Solyc12g014220.1.1 | 2,37 | -2,551 | Homology to unknown gene (Fragment) |
| Solyc12g014620.1.1 | 2,281 | -2,739 | Cortical cell-delineating protein |
| Solyc04g080730.2.1 | 1,49 | -3,074 | Mitogen-activated protein kinase 9 |
| Solyc12g096060.1.1 | 2,117 | -3,071 | Mps one binder kinase activator-like 1A |
| Solyc01g095320.2.1 | 2,02 | -3,128 | BCL-2-associated athanogene 6 |
| Solyc05g053210.2.1 | 3,672 | -6,171 | CBL-interacting protein kinase 1 |
| Solyc04g078820.2.1 | -1,709 | 2,534 | Annexin |
| Solyc02g093420.2.1 | 2,26 | -4,177 | NAC domain class transcription factor |
| Solyc05g032660.2.1 | 2,047 | -2,381 | Dehydrogenase/ reductase 3 |
