# Supplementary material for: Modulation of Tomato Response to Rhizoctonia solani by Trichoderma harzianum and Its Secondary Metabolite Harzianic Acid
Source: Front Microbiol. 2018 Aug 30;9:1966. doi: 10.3389/fmicb.2018.01966 (PMC6127634; doi:10.3389/fmicb.2018.01966)
Supplement: Supplementary file 3 [file Table_3.DOCX]

**Table S3: List of shared up-regulated DEGs found in the comparative analysis [P+HA+R *vs.* P+R] *vs.* [P+T+R *vs*. P+R] *vs*. [P+T *vs.* P].** **A**: genes up-regulated in [P+T *vs.* P] and [P+HA+R *vs.* P+R] and down-regulated in [P+T+R *vs.* P+R]; **B**: genes up-regulated only in infected plants [P+T+R *vs.* P+R] and [P+HA+R *vs.* P+R] and down regulated in non-infected plants [P+T *vs.* P]; **C:** genes up-regulated in [P+T *vs.* P] and down-regulated in the multiple interaction ([P+HA+R *vs.* P+R] and [P+T+R *vs.* P+R]). [P+T *vs.* P]: Plant treated with *Trichoderma*; [P+T+R *vs.* P+R]: *R.solani* infected plants treated with *Trichoderma*; ]P+HA+R *vs.* P+R]: *R.solani* infected plants treated with HA.

|  | Treatment | ID | annotation |
| --- | --- | --- | --- |
| **A** | [P+T vs P] and [P+HA+R vs P+R]  **6 genes** | Solyc10g084400.1.1 | "Glutathione S-transferase |
|  |  | Solyc02g083810.2.1 | Ferredoxin--NADP reductase |
|  |  | Solyc08g074560.2.1 | Uncharacterized aarF domain-containing protein kinase 1 |
|  |  | Solyc07g062140.2.1 | Alpha alpha-trehalose-phosphate synthase |
|  |  | Solyc05g053210.2.1 | CBL-interacting protein kinase 1 |
|  |  | Solyc08g075700.2.1 | 60S ribosomal protein L13 |
| **B** | [P+T+R vs P+R] and [P+HA+R vs P+R]  **12 genes** | Solyc08g023660.2.1 | "Major latex-like protein |
|  |  | Solyc02g080760.1.1 | Glycine-rich protein |
|  |  | Solyc06g049050.2.1 | "Expansin |
|  |  | Solyc04g077020.2.1 | Tubulin alpha-3 chain |
|  |  | Solyc01g007380.1.1 | GO:0031361 "Apocytochrome f |
|  |  | Solyc10g005730.2.1 | WD-40 repeat family protein |
|  |  | Solyc08g074620.1.1 | Polyphenol oxidase |
|  |  | Solyc04g005700.2.1 | Major latex-like protein |
|  |  | Solyc07g063430.2.1 | Mpv17 protein |
|  |  | Solyc03g115900.2.1 | Chlorophyll a-b binding protein P4, chloroplastic |
|  |  | Solyc01g007330.2.1 | Ribulose bisphosphate carboxylase large chain |
|  |  | Solyc10g006900.2.1 | "Protochlorophyllide reductase |
| **C** | [P+T vs P]  **68 genes**  [P+T vs P] | Solyc01g010660.2.1 | Receptor-like protein kinase At3g21340 |
|  |  | Solyc01g068150.2.1 | Chromosome 11 contig 1 DNA sequence |
|  |  | Solyc06g082280.2.1 | Serine-threonine protein phosphatase |
|  |  | Solyc02g088780.2.1 | Ribosome biogenesis protein ytm1 |
|  |  | Solyc01g098230.2.1 | Mitochondrial import inner membrane translocase subunit TIM44 |
|  |  | Solyc12g096060.1.1 | Mps one binder kinase activator-like 1A |
|  |  | Solyc07g066330.2.1 | NAC domain protein IPR003441 |
|  |  | Solyc04g007160.1.1 | Alpha-glucosidase |
|  |  | Solyc03g114920.1.1 | Pentatricopeptide repeat-containing protein |
|  |  | Solyc03g044060.2.1 | Formin 3 |
|  |  | Solyc09g092390.2.1 | "Adenosylhomocysteinase |
|  |  | Solyc11g008250.1.1 | Single-stranded nucleic acid binding R3H domain protein |
|  |  | Solyc10g062180.1.1 | Polyadenylate-binding protein |
|  |  | Solyc09g075440.2.1 | Ethylene receptor |
|  |  | Solyc06g071470.2.1 | Peroxisomal membrane protein PEX14 |
|  |  | Solyc02g092240.2.1 | Os10g0352000 protein (Fragment) |
|  |  | Solyc08g007240.2.1 | Hydrolase NUDIX family protein |
|  |  | Solyc01g087680.2.1 | Uncharacterized membrane protein |
|  |  | Solyc06g075110.2.1 | Lysine ketoglutarate reductase trans-splicing related 1 |
|  |  | Solyc11g044320.1.1 | Single myb histone |
|  |  | Solyc09g065900.2.1 | Glutathione reductase |
|  |  | Solyc10g080720.1.1 | Polypyrimidine tract binding protein 1 |
|  |  | Solyc09g011480.2.1 | Rop guanine nucleotide exchange factor 1 |
|  |  | Solyc12g014220.1.1 | Homology to unknown gene (Fragment) |
|  |  | Solyc02g082070.2.1 | Cytochrome P450 |
|  |  | Solyc11g017140.1.1 | C2-H2 zinc finger protein |
|  |  | Solyc09g075000.2.1 | WD repeat protein |
|  |  | Solyc03g111730.2.1 | Cathepsin B-like cysteine proteinase |
|  |  | Solyc01g079760.2.1 | Mitochondrial carrier protein |
|  |  | Solyc02g079590.2.1 | Serine/threonine kinase receptor |
|  |  | Solyc05g049950.2.1 | Small nuclear ribonucleoprotein-associated protein B |
|  |  | Solyc12g014620.1.1 | "Cortical cell-delineating protein |
|  |  | Solyc02g090970.1.1 | Serine/threonine-protein kinase 24 |
|  |  | Solyc02g092920.2.1 | Cadmium-transporting ATPase |
|  |  | Solyc03g078520.2.1 | Receptor like kinase, RLK |
|  |  | Solyc01g080540.2.1 | "Histidine-containing phosphotransfer protein |
|  |  | Solyc06g065820.2.1 | Ethylene-responsive transcription factor 7 |
|  |  | Solyc01g080090.2.1 | Zinc finger RING-type protein |
|  |  | Solyc11g062130.1.1 | "Mitochondrial ADP/ATP carrier |
|  |  | Solyc02g093420.2.1 | "NAC domain class transcription factor |
|  |  | Solyc05g056010.2.1 | Serine/threonine-protein phosphatase (Fragment) |
|  |  | Solyc02g021760.2.1 | mRNA 3&apos-end-processing protein yth1 |
|  |  | Solyc06g008310.2.1 | Elongator complex protein 2 |
|  |  | Solyc05g032660.2.1 | Dehydrogenase/ reductase 3 |
|  |  | Solyc10g007120.2.1 | Genomic DNA chromosome 3 BAC clone F4B12 |
|  |  | Solyc10g017530.2.1 | "ATP-dependent RNA Helicase |
|  |  | Solyc02g077330.2.1 | GDSL esterase/lipase At5g45950 |
|  |  | Solyc10g039280.1.1 | Dual specificity protein phosphatase family protein |
|  |  | Solyc03g115110.2.1 | ATP synthase gamma chain |
|  |  | Solyc01g010870.2.1 | Dehydration-responsive family protein |
|  |  | Solyc02g085210.1.1 | UPF0567 protein C13orf39 homolog |
|  |  | Solyc08g081480.2.1 | Polygalacturonase-like protein |
|  |  | Solyc12g042540.1.1 | "Unknown Protein |
|  |  | Solyc03g007670.2.1 | SGT1 |
|  |  | Solyc03g116890.2.1 | "WRKY transcription factor 2 |
|  |  | Solyc03g082960.2.1 | Serine/threonine phosphatase family protein |
|  |  | Solyc10g005590.2.1 | Nuclear cap-binding protein subunit 2 |
|  |  | Solyc02g037550.2.1 | Auxin efflux carrier family protein |
|  |  | Solyc07g064940.2.1 | Thioredoxin family protein |
|  |  | Solyc04g040200.2.1 | Cellular retinaldehyde-binding/triple function C-terminal |
|  |  | Solyc11g011040.1.1 | ADP-ribosylation factor |
|  |  | Solyc09g089580.2.1 | 1-aminocyclopropane-1-carboxylate oxidase-like protein |
|  |  | Solyc07g064930.2.1 | "Protein grpE |
|  |  | Solyc06g065440.1.1 | Zinc finger family protein |
|  |  | Solyc02g092680.1.1 | Subtilisin-like protease |
|  |  | Solyc11g006690.1.1 | GO:0005840 "40S ribosomal protein S17-like protein |
|  |  | Solyc01g107250.2.1 | LRR receptor-like serine/threonine-protein kinase, RLP |
|  |  | Solyc04g080730.2.1 | Mitogen-activated protein kinase 9 |
